# A Neutral Beryllium(I) Radical

**DOI:** 10.1002/anie.202108405

**Published:** 2021-08-24

**Authors:** Corinna Czernetzki, Merle Arrowsmith, Felipe Fantuzzi, Annalena Gärtner, Tobias Tröster, Ivo Krummenacher, Fabian Schorr, Holger Braunschweig

**Affiliations:** ^1^ Institute for Inorganic Chemistry Julius-Maximilians-Universität Würzburg Am Hubland 97074 Würzburg Germany; ^2^ Institute for Sustainable Chemistry & Catalysis with Boron Julius-Maximilians-Universität Würzburg Am Hubland 97074 Würzburg Germany

**Keywords:** Beryllium, cyclic alkyl(amino)carbene, EDA-NOCV, radical, X-ray crystallography

## Abstract

The reduction of a cyclic alkyl(amino)carbene (CAAC)‐stabilized organoberyllium chloride yields the first neutral beryllium radical, which was characterized by EPR, IR, and UV/Vis spectroscopy, X‐ray crystallography, and DFT calculations.

## Graphical abstract

The reduction of a cyclic alkyl(amino)carbene (CAAC)‐stabilized organoberyllium chloride yields the first neutral beryllium radical, which was characterized by EPR, IR, UV/Vis spectroscopy and X‐ray crystallography. DFT calculations show significant spin density at beryllium and confirm donor–acceptor bonding between an alkylberyllium radical fragment and a neutral CAAC ligand.

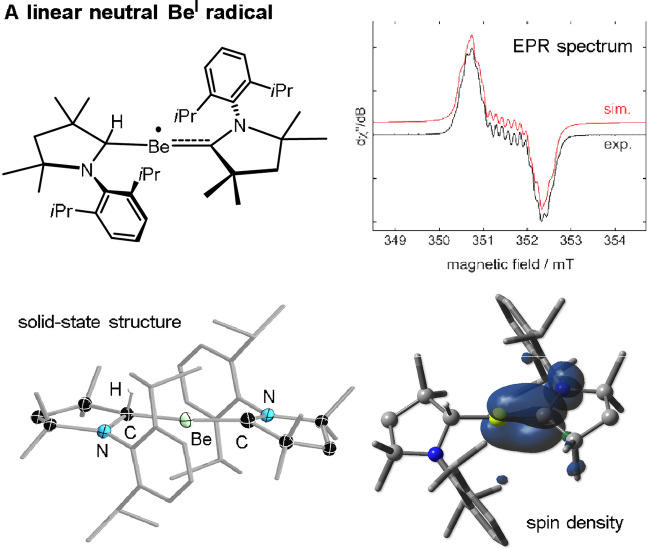

While group 2 chemistry is mainly dictated by the naturally occurring +2 oxidation state of its elements, the last two decades have seen the emergence of a growing number of low‐oxidation‐state alkaline earth metal compounds. Since the landmark synthesis of the first dinuclear Mg^I^ complexes, including **I** (Figure 1),^[1]^ these compounds have been successfully applied as highly selective reducing agents for the activation of small molecules^[2]^ and the synthesis of new homo‐ and heteronuclear metal−metal bonds,^[3]^ culminating most recently in the isolation of the first molecular Mg^0^ species, complex **II**.^[4]^

**Figure 1:**
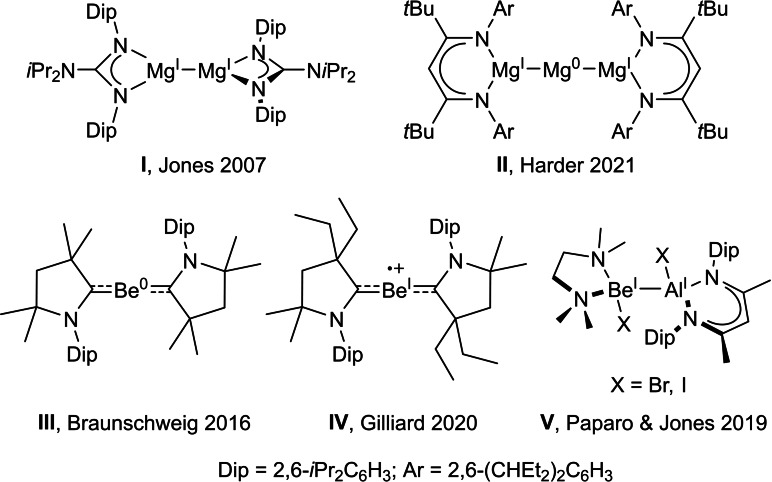
Selection of low‐oxidation‐state molecular group 2 complexes.

Low‐valent beryllium complexes long remained confined to the computational realm due to their very high toxicity. Recent years, however, have seen a renewed interest in beryllium coordination chemistry in the areas of organometallic, pure inorganic, and bioinorganic chemistry.^[5]^ Although the viability of Be^I^−Be^I^‐bonded species has been predicted,^[6]^ and the Be_2_ dimer has been observed spectroscopically,^[7]^ the low Be−Be bond enthalpy makes mononuclear Be^0^ compounds more accessible targets.^[8]^ In 2016 our group reported the first Be^0^ compound, complex **III**, which owes its stability to strong three‐center‐two‐electron π backbonding from the Be^0^ atom in its 2s^0^2p^2^ electronic configuration to the neutral cyclic alkyl(amino)carbene (CAAC) ligands,^[9]^ and this has since been used as a reducing agent to synthesize the first carbene bismuthinidene complex.^[10]^ Although beryllium radicals have been postulated as intermediates in reduction reactions resulting in ligand activation,^[11]^ the first isolable Be^I^ radical cation, **IV**, was only reported in 2020 from the one‐electron oxidation of an analogue of **III** with 2,2,6,6‐tetramethylpiperidin‐1‐oxyl.^[12]^ Calculations showed that the bonding in **IV** is similar to that in **III**, with two neutral CAAC ligands stabilizing a Be^I^ cation in its excited 2s^0^2p^1^ electronic configuration through donor–acceptor interactions, and that the spin density is delocalized over the entire N‐C‐Be‐C‐N framework, with 38 % located at the beryllium center. Furthermore, Paparo and Jones succeeded in isolating the first neutral Be^I^ complexes, such as **V**, which present covalent Be^I^−Al^I^ bonding.^[13]^ We now report the synthesis and computational analysis of the first structurally characterized neutral beryllium radical, stabilized by both a neutral and a C1‐protonated CAAC ligand.

The organoberyllium halide precursors (CAAC)(CAACH)BeX (CAAC=1‐(2,6‐diisopropylphenyl)‐3,3,5,5‐tetramethylpyrrolidin‐2‐ylidene; CAACH=1‐(2,6‐diisopropylphenyl)‐3,3,5,5‐tetramethylpyrrolidin‐2‐yl; X=Cl, Br) were synthesized by the addition of L‐selectride (Li[HB*s*Bu_3_]) to a 1:1 mixture of CAAC and (CAAC)BeX_2_ ^[9]^ in toluene at −78 °C and isolated as pale orange solids in excellent yields (≥82 %, Scheme 1 a). The reaction likely proceeds via formation of a (CAAC)Be(H)Cl intermediate, followed by coordination of the second CAAC ligand to form (CAAC)_2_Be(H)Cl, and finally a 1,2‐hydride shift from the beryllium center to the CAAC carbene carbon atom. Such 1,2‐hydrogen shifts are common in CAAC‐stabilized main group hydrides upon coordination of an additional Lewis base.^[14]^ A characteristic ^1^H singlet around 3.0 ppm and the complex ligand resonance patterns of the ^1^H NMR spectra confirmed the protonation of one of the CAAC ligands. The ^9^Be NMR shifts of the two complexes appear at 19 and 20 ppm for X=Cl and Br, respectively. In order to place the ^9^Be NMR shifts of (CAAC)(CAACH)BeX in context with comparable CAAC‐stabilized tricoordinate beryllium complexes, they are downfield‐shifted from those of their (CAAC)BeX_2_ precursors at 12.9 and 14.0 ppm, respectively,^[9]^ slightly upfield‐shifted from that of the beryllole (CAAC)BeC_4_Ph_4_ at 22.9 ppm,^[15]^ and similar to that of the diazaborolyl beryllium chloride (CAAC)BeCl(B(NDipCH)_2_) at 20 ppm.^[16]^ Additionally, the complexes were characterized by X‐ray crystallographic analyses (see Figures S19 and S21 in the Supporting Information).^[25]^ Cyclic voltammetry (CV) experiments carried out in difluorobenzene showed a single irreversible reduction wave at *E*_pc_=−1.83 V versus the ferrocene/ferrocenium couple (Fc/Fc^+^) for (CAAC)(CAACH)BeCl, whereas (CAAC)(CAACH)BeBr showed two irreversible reduction waves at *E*_pc_=−1.85 V and −2.46 V, which hint at the potential for chemical reduction of both species.

**Scheme 1:**
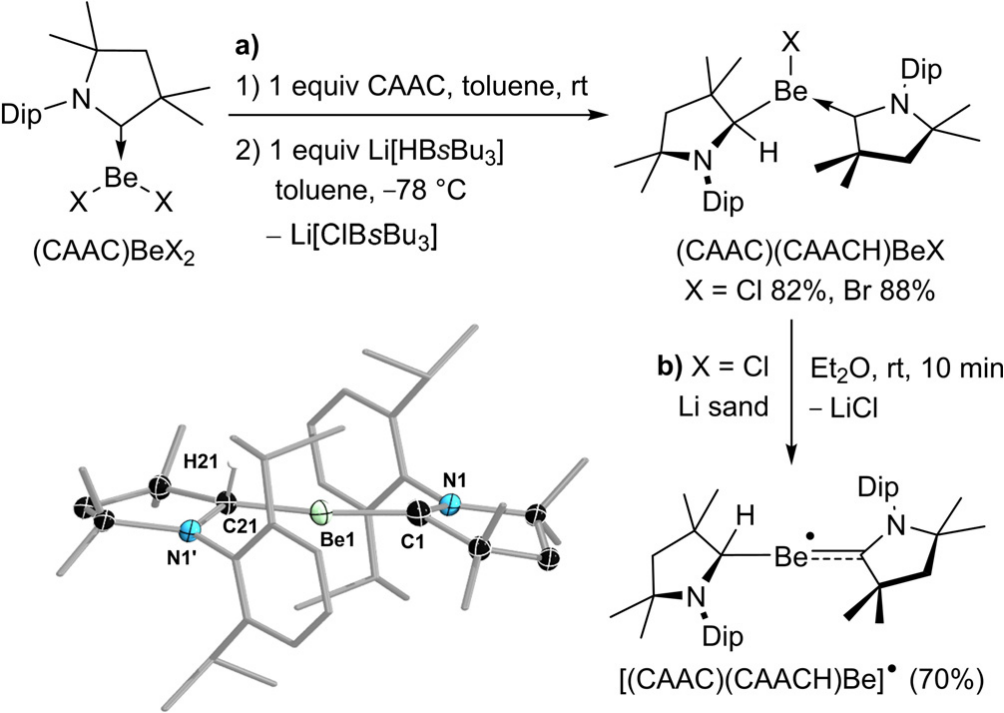
Synthesis and crystallographically derived solid‐state structure of [(CAAC)(CAACH)Be]^.^ (only the major part of the two flip‐disordered Be(CAACH) units shown). Atomic displacement ellipsoids represented at 50 % and omitted for the ligand periphery. Hydrogen atoms omitted, except for H21. Selected bond lengths (Å) and angles (°): N1‐C1 1.321(2), C1‐Be1 1.661(7), Be1‐C21 1.730(7), C21‐N1′ 1.548(4); C1‐Be1‐C21 170.7(3).^[25]^

Whereas the reduction of (CAAC)(CAACH)BeBr with a wide range of reducing agents in various solvents at best resulted in partial reduction and the formation of [CAACH]Br as the sole isolable product, the room‐temperature reduction of (CAAC)(CAACH)BeCl with lithium sand in diethyl ether over a period of 10 minutes resulted in the formation of the radical species [(CAAC)(CAACH)Be]^.^, which was isolated as a brown‐orange crystalline solid in 70 % yield (Scheme 1 b). While the radical proved stable in the solid state at −30 °C under an argon atmosphere for several weeks, it decomposed within minutes in polar solvents, such as THF and 1,2‐difluorobenzene, and within two days in diethyl ether at −30 °C. In aromatic hydrocarbon solvents, such as benzene and toluene, the compound was less soluble but remained stable at room temperature, provided silanized glassware or polyethylene vials were used to avoid its reaction with glassware surface OH groups. Under these conditions [(CAAC)(CAACH)Be]^.^ could be heated up to 60 °C before significant decomposition set in.

As expected, [(CAAC)(CAACH)Be]^.^ was NMR‐silent but displayed a complex EPR signal centered at *g*_iso_=2.003. Simulation provided a hyperfine coupling constant to ^9^Be of 11.6 MHz (4.1 G, Figure 2 a), significantly larger than for **IV** (0.32 G).^[12]^ Calculations at the UBP86‐D3(BJ)/def2SVP level of theory, performed using Gaussian 16,^[17]^ show that the SOMO is mainly delocalized over the Be−C_CAAC_ π bond, with some π‐antibonding character on the C−N bond of the CAAC ligand (Figure 2 b), as is the case for most CAAC‐stabilized main group radicals.^[18]^ The calculated spin density at beryllium (23 %) is significantly lower than that calculated for the radical cation **IV** (38 %).[^12^, ^19^]

**Figure 2:**
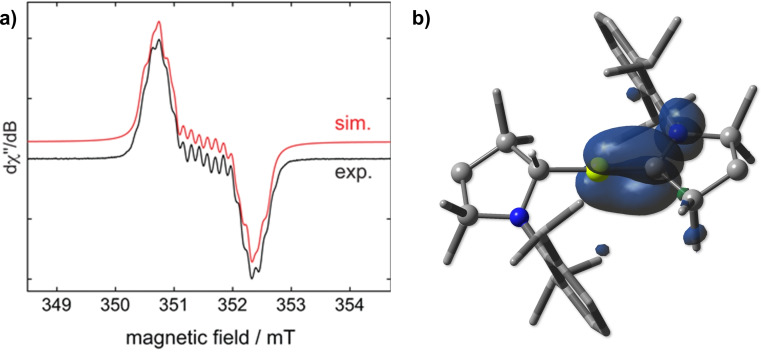
a) Experimental (black) and simulated (red) continuous‐wave (CW) X‐band EPR spectra of [(CAAC)(CAACH)Be]^.^ in benzene at room temperature. The simulation parameters are *g*_iso_=2.003, *a*(^9^Be)= 11.6 MHz, *a*(^14^N)=3.7 MHz, and *a*(^1^H)=6.3 MHz. b) Plot of spin density of [(CAAC)(CAACH)Be]^.^ calculated at the UBP86‐D3(BJ)/def2SVP level of theory. Mulliken spin densities: 0.23 (Be1); 0.54 (C1); 0.19 (N1).

The solid‐state IR spectrum of [(CAAC)(CAACH)Be]^.^ shows a characteristic band at 2693 cm^−1^ which calculations attribute to the C−H stretching frequency of the protonated beryllium‐bound carbon atom ($\tilde{\nu }$_calcd_=2725 cm^−1^). The UV/Vis spectrum of [(CAAC)(CAACH)Be]^.^, which had to be recorded in Et_2_O in a silanized cuvette to avoid decomposition, shows a broad absorption centered at *λ*_max_=350 nm, spanning over 100 nm at mid‐height and extending into the 400–500 nm range, thus accounting for the brown‐orange coloration of the radical.^[20]^ Accordingly, TD‐DFT calculations indicate the presence of charge‐transfer transitions from the SOMO to low‐lying LUMOs in this wavelength window, the one with the largest oscillator strength appearing at 330 nm (UCAM‐B3LYP/6‐31++G**, see Supporting Information).

Independent of the crystallization conditions [(CAAC)(CAACH)Be]^.^ crystallized in the *P* $\bar{1}$ space group (see solid‐state structure in Scheme 1), with one fully centrosymmetrically disordered molecule per asymmetric unit, which further presents a twofold disorder in the relative *R*/*S* configuration of the CAACH ligand backbone in a ca. 4:1 ratio.^[21]^ While the structural data may not, therefore, be discussed in detail, it confirms that the beryllium center displays a near‐linear geometry (C1‐B1‐C21 ca. 171°) and that the Be−C1 bond to the neutral CAAC ligand (ca. 1.66 Å) is significantly shorter than the Be1−C21 single bond to the protonated CAACH ligand (ca. 1.73 Å). The partial double bond character of C1−Be1 is also supported by Mayer bond order calculations, the value of which (1.10) is significantly larger than that of Be1−C21 (0.79).

The energy decomposition analysis in combination with the natural orbitals for chemical valence method (EDA‐NOCV), as implemented in ADF 2019,^[22]^ was applied to [(CAAC′)(CAAC′H)Be]^.^ (truncated model with Me and *i*Pr groups replaced by hydrogen atoms) in order to investigate its bonding situation. The results were obtained at the BP86‐D3(BJ)/TZV2P level of theory. The quantitative results for three distinct decomposition schemes are shown in Table S1 in the Supporting Information. These were based on [(CAAC′H)Be]^.^ and CAAC′ as interacting fragments and varied depending on the electronic configuration and multiplicity of the fragments. The interaction between [(CAAC′H)Be]^.^ in its first excited doublet configuration, where the radical occupies a p_⊥_ orbital of Be, and a ground‐state singlet CAAC′ resulted in the lowest orbital interaction term Δ*E*_orb_. As this is a useful criterion for discerning the best bonding description in terms of interacting fragments,[^15^, ^23^] we conclude that donor–acceptor interactions are at play in the stabilization of the [(CAAC′)(CAAC′H)Be]^.^ neutral radical.

As shown in Table S1 in the Supporting Information, essentially half of the attraction (50.3 %) between the [(CAAC′H)Be]^.^ and CAAC′ fragments is due to the covalent contribution Δ*E*_orb_. The dispersion contribution (Δ*E*_disp_) accounts for merely 2.6 % and the electrostatic attraction Δ*E*_elstat_ is responsible for the remaining 47.1 %. These results are comparable to those observed for the paramagnetic beryllium radical cation **IV**.^[12]^ The breakdown of Δ*E*_orb_ into pairwise orbital interactions (Table SX and Figure 3) shows that the strongest contribution comes from the (CAAC′H)Be→CAAC′ π backdonation from the Be radical into the vacant π orbital of the CAAC′ ligand (Δ*E*_orb(2α)_=−56.5 kcal mol^−1^, see Figure 3 for the corresponding deformation density). This contribution is slightly stronger than that obtained for the radical cation **IV** ^[12]^ and significantly weaker than that of the neutral Be^0^ species **III**,^[9]^ the latter on account of the half‐empty Be p_⊥_ orbital of [(CAAC′)(CAAC′H)Be]^.^, which is a weaker donor in comparison to the doubly occupied Be p_⊥_ orbital of **III**. In contrast, the (CAAC′H)Be←CAAC′ σ donation (see Figure 3 for the corresponding deformation density) is Δ*E*_orb(1αβ)_=−43.4 kcal mol^−1^, weaker than the CAAC→Be←CAAC σ donation contributions in **III** and **IV**. This is explained by the fact that in this case only one CAAC ligand contributes to the σ donation, while in the previous cases both ligands donate to the central Be atom.

**Figure 3:**
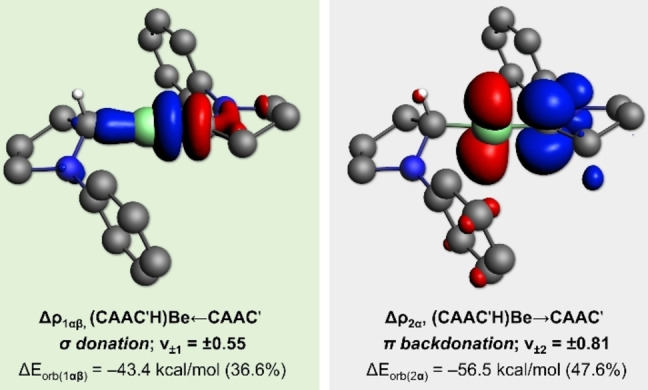
Plots of the deformation densities Δ*ρ*_1αβ_ and Δ*ρ*_2α_ of the main pairwise contributions associated with the orbital interaction term Δ*E*_orb_ in the truncated model [(CAAC′)(CAAC′H)Be]^.^. Interacting fragments: [(CAAC′H)Be]^.^ (radical in the p_⊥_ orbital of Be) and CAAC′ (closed‐shell singlet ground state). Values in parentheses are the percentage of the pairwise orbital interaction with respect to the total Δ*E*_orb_ contribution. The *ν*_±*k*_ values correspond to the eigenvalues of the complementary eigenfunctions (*ψ*_−*k*_, *ψ*_+*k*_) in the NOCV representation. Isovalues: 0.003. Charge flows from red to blue.

In order to assess the possibility of fluxional hydrogen shifting from CAACH to CAAC in [(CAAC)(CAACH)Be]^.^ via an intermediate tricoordinate tautomer [(CAAC)_2_BeH]^.^, we also examined the latter computationally (Figure 4). At the UBP86‐D3(BJ)/def2SVP level of theory [(CAAC)_2_BeH]^.^ lies 10.5 kcal mol^−1^ higher in energy than [(CAAC)(CAACH)Be]^.^. Its SOMO is π‐delocalized along the two CAAC ligands and the central Be atom and features two nodal planes at the C−N bonds. In contrast, the LUMO of [(CAAC)_2_BeH]^.^, which is mostly located at the endocyclic C−N bonds, is stabilized by 0.56 eV compared to that of [(CAAC)(CAACH)Be]^.^. This leads to a decrease in the SOMO–LUMO (SL) gap of [(CAAC)_2_BeH]^.^ to merely 0.75 eV, less than half the SL gap of [(CAAC)(CAACH)Be]^.^ (1.61 eV). These results show that a doubly CAAC‐stabilized BeH radical is not energetically accessible. Since the nature of the Lewis base (L) strongly influences the electronic and structural features of main group compounds,^[24]^ a theoretical investigation of various [L_2_BeH]^.^ radicals, aiming at the identification of potential synthetic targets, is currently under investigation in our group.

**Figure 4:**
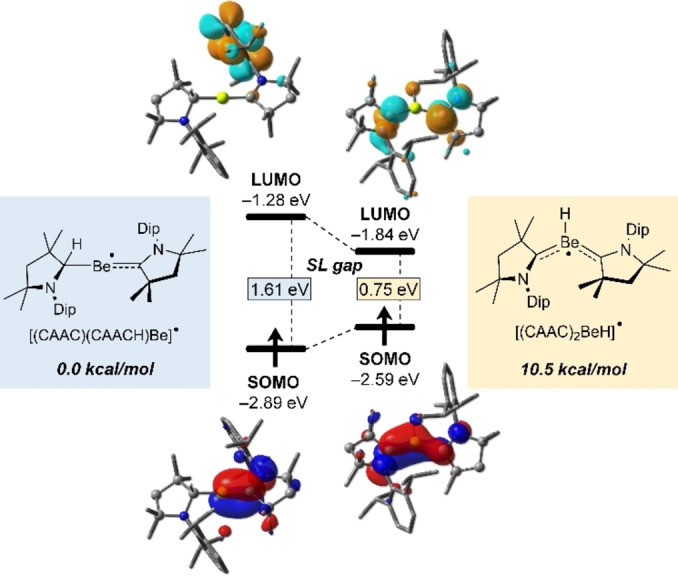
Canonical Kohn–Sham molecular orbitals of [(CAAC)(CAACH)Be]^.^ and its putative tautomer [(CAAC)_2_BeH]^.^ at the UBP86‐D3(BJ)/def2‐SVP level of theory.

To summarize, we have synthesized and structurally characterized a stable neutral Be^I^ radical, the first example of an isolable neutral s‐block radical with significant spin density located at the metal center. We are currently investigating the reactivity of this species and will report our findings in due course.

## Conflict of interest

The authors declare no conflict of interest.

## Supporting information

As a service to our authors and readers, this journal provides supporting information supplied by the authors. Such materials are peer reviewed and may be re‐organized for online delivery, but are not copy‐edited or typeset. Technical support issues arising from supporting information (other than missing files) should be addressed to the authors.

Supporting Information

## References

[1] S. P. Green , C. Jones , A. Stasch , Science 2007, 318, 1754–1757. 17991827 10.1126/science.1150856

[2] Selected examples:

[2a] J. Krüger , C. Wölper , S. Schulz , Inorg. Chem. 2020, 59, 11142–11151; 32663023 10.1021/acs.inorgchem.0c01657

[2b] C. Bakewell , B. J. Ward , A. J. P. White , M. R. Crimmin , Chem. Sci. 2018, 9, 2348–2356; 29719707 10.1039/c7sc05059c PMC5897846

[2c] C. Ganesamoorthy , C. Wölper , A. S. Nizovtsev , S. Schulz , Angew. Chem. Int. Ed. 2016, 55, 4204–4209; 10.1002/anie.201510504 26924273

[2d] J. Hicks , E. J. Underhill , C. E. Kefalidis , L. Maron , C. Jones , Angew. Chem. Int. Ed. 2015, 54, 10000–10004; 10.1002/anie.201504818 26126428

[2e] J. Hicks , C. E. Hoyer , B. Moubaraki , G. Li Manni , E. Carter , D. M. Murphy , K. S. Murray , L. Gagliardi , C. Jones , J. Am. Chem. Soc. 2014, 136, 5283–5286. 24660853 10.1021/ja5021348

[3] Selected recent examples:

[3a] A. Paparo , K. Yuvaraj , A. J. R. Matthews , I. Douair , L. Maron , C. Jones , Angew. Chem. Int. Ed. 2021, 60, 630–634; 10.1002/anie.202009523 32969564

[3b] R. Y. Kong , J. Am. Chem. Soc. 2020, 142, 11967–11971; 32589418 10.1021/jacs.0c03383

[3c] K. Yuvaraj , I. Douair , A. Paparo , L. Maron , C. Jones , J. Am. Chem. Soc. 2019, 141, 8764–8768; 31096751 10.1021/jacs.9b04085

[3d] D. Dange , A. R. Gair , D. D. L. Jones , M. Juckel , S. Aldridge , C. Jones , Chem. Sci. 2019, 10, 3208–3216; 30996903 10.1039/c9sc00200f PMC6428033

[3e] A. J. Boutland , A. Carroll , C. Alvarez Lamsfus , A. Stasch , L. Maron , C. Jones , J. Am. Chem. Soc. 2017, 139, 18190–18193; 29206455 10.1021/jacs.7b11368

[3f] R. Lalrempuia , C. E. Kefalidis , S. J. Bonyhady , B. Schwarze , L. Maron , A. Stasch , C. Jones , J. Am. Chem. Soc. 2015, 137, 8944–8947; 26135846 10.1021/jacs.5b06439

[3g] C. Bakewell , A. J. P. White , M. R. Crimmin , J. Am. Chem. Soc. 2016, 138, 12763–12766. 27636244 10.1021/jacs.6b08104 PMC5135227

[4] B. Rösch , T. X. Gentner , J. Eyselein , J. Langer , H. Elsen , S. Harder , Nature 2021, 592, 717–721. 33911274 10.1038/s41586-021-03401-w

[5] Selected reviews:

[5a] J. K. Buchanan , P. G. Plieger , Chem. Lett. 2021, 50, 227–234;

[5b] M. R. Buchner , Chem. Commun. 2020, 56, 8895–8907; 10.1039/d0cc03802d 32578609

[5c] L. C. Perera , O. Raymond , W. Henderson , P. J. Brothers , P. G. Plieger , Coord. Chem. Rev. 2017, 352, 264–290;

[5d] D. Naglav , M. R. Buchner , G. Bendt , F. Kraus , S. Schulz , Angew. Chem. Int. Ed. 2016, 55, 10562–10576; 10.1002/anie.201601809 27364901

[5e] K. J. Iversen , S. A. Couchman , D. J. D. Wilson , J. L. Dutton , Coord. Chem. Rev. 2015, 297–298, 40–48;

[5f] R. Puchta , Nat. Chem. 2011, 3, 416; 21505503 10.1038/nchem.1033

[5g] K. Dehnicke , B. Neumüller , Z. Anorg. Allg. Chem. 2008, 634, 2703–2728.

[6a] X. Liu , M. Zhang , R. Zhong , S. Wu , Y. Liu , Y. Geng , Z. Su , Chem. Eur. J. 2020, 26, 10891–10895; 32297691 10.1002/chem.201905230

[6b] Z.-Z. Qin , Q. Wang , C. Yuan , Y.-T. Yang , X.-F. Zhao , D. Li , P. Liu , Y.-B. Wu , Dalton Trans. 2018, 47, 4707–4713; 29537009 10.1039/c7dt04897a

[6c] C. Yuan , X.-F. Zhao , Y.-B. Wu , X. Wang , Angew. Chem. Int. Ed. 2016, 55, 15651–15655; 10.1002/anie.201609455 27860145

[6d] A. Baishya , V. R. Mundlapati , S. Nembenna , H. S. Biswal , J. Chem. Sci. 2014, 126, 1781–1788.

[7a] J. M. Merritt , V. E. Bondybey , M. C. Heaven , Science 2009, 324, 1548–1551; 19460963 10.1126/science.1174326

[7b] V. E. Bondbey , Science 1985, 227, 125–131. 17843064 10.1126/science.227.4683.125

[8a] A. J. Kalita , S. S. Rohman , C. Kashyap , S. S. Ullah , L. J. Mazumder , A. K. Guha , ChemistrySelect 2020, 5, 8798–8805;

[8b] S. A. Couchmann , N. Holzmann , G. Frenking , D. J. D. Wilson , J. L. Dutton , Dalton Trans. 2013, 42, 11375–11384; 23572069 10.1039/c3dt50563d

[8c] S. De , P. Parameswaran , Dalton Trans. 2013, 42, 4650–4656. 23360926 10.1039/c3dt32749c

[9] M. Arrowsmith , H. Braunschweig , M. A. Celik , T. Dellermann , R. D. Dewhurst , W. C. Ewing , K. Hammond , T. Kramer , I. Krummenacher , J. Mies , K. Radacki , J. K. Schuster , Nat. Chem. 2016, 8, 890–894. 10.1038/nchem.2542 27334631

[10] G. Wang , L. A. Freeman , D. A. Dickie , R. Mokrai , Z. Benkő , R. J. Gilliard , Chem. Eur. J. 2019, 25, 4335–4339. 30706565 10.1002/chem.201900458 PMC6593863

[11a] J. E. Walley , G. Breiner , G. Wang , D. A. Dickie , A. Molino , J. L. Dutton , D. J. D. Wilson , R. J. Gilliard , Chem. Commun. 2019, 55, 1967–1970; 10.1039/c8cc10022e 30681680

[11b] M. Arrowsmith , M. S. Hill , G. Kociok-Köhn , D. J. MacDougall , M. F. Mahon , I. Mallov , Inorg. Chem. 2012, 51, 13408–13418. 23215345 10.1021/ic3022968

[12] G. Wang , J. E. Walley , D. A. Dickie , S. Pan , G. Frenking , R. J. Gilliard , J. Am. Chem. Soc. 2020, 142, 4560–4564. 32088963 10.1021/jacs.9b13777

[13a] A. Paparo , A. J. R. Matthews , C. D. Smith , A. Edwards , K. Yuvaraja , C. Jones , Dalton Trans. 2021, 50, 7604–7609; 33988210 10.1039/d1dt01393a

[13b] A. Paparo , C. D. Smith , C. Jones , Angew. Chem. Int. Ed. 2019, 58, 11459–11463; 10.1002/anie.201906609 31206958

[14a] A. Hock , L. Werner , C. Luz , U. Radius , Dalton Trans. 2020, 49, 11108–11119; 32743616 10.1039/d0dt02070b

[14b] S. Hagspiel , M. Arrowsmith , F. Fantuzzi , A. Hermann , V. Paprocki , R. Drescher , I. Krummenacher , H. Braunschweig , Chem. Sci. 2020, 11, 551–555; 32206272 10.1039/c9sc05026d PMC7069503

[14c] S. K. Mellerup , Y. Cui , F. Fantuzzi , P. Schmid , J. T. Goettel , G. Bélanger-Chabot , M. Arrowsmith , I. Krummenacher , Q. Ye , V. Engel , B. Engels , H. Braunschweig , J. Am. Chem. Soc. 2019, 141, 16954–16960; 31577138 10.1021/jacs.9b09128

[14d] L. L. Cao , D. W. Stephan , Chem. Commun. 2018, 54, 8407–8410; 10.1039/c8cc05013a 29998266

[14e] H. Schneider , A. Hock , R. Bertermann , U. Radius , Chem. Eur. J. 2017, 23, 12387–12398; 28603878 10.1002/chem.201702166

[14f] D. Auerhammer , M. Arrowsmith , H. Braunschweig , R. D. Dewhurst , J. O. C. Jiménez-Halla , T. Kupfer , Chem. Sci. 2017, 8, 7066–7071. 29147534 10.1039/c7sc03193a PMC5637459

[15] D. K. Roy , T. Tröster , F. Fantuzzi , R. D. Dewhurst , C. Lenczyk , K. Radacki , C. Pranckevicius , B. Engels , H. Braunschweig , Angew. Chem. Int. Ed. 2021, 60, 3812–3819; 10.1002/anie.202014557 PMC7898526 33210400

[16] J. K. Schuster , D. K. Roy , C. Lenczyk , J. Mies , H. Braunschweig , Inorg. Chem. 2019, 58, 2652–2658. 30707568 10.1021/acs.inorgchem.8b03263

[17] M. J. Frisch, G. W. Trucks, H. B. Schlegel, G. E. Scuseria, M. A. Robb, J. R. Cheeseman, G. Scalmani, V. Barone, B. Mennucci, G. A. Petersson, H. Nakatsuji, M. Caricato, X. Li, H. P. Hratchian, A. F. Izmaylov, J. Bloino, G. Zheng, J. L. Sonnenberg, M. Hada, M. Ehara, K. Toyota, R. Fukuda, J. Hasegawa, M. Ishida, T. Nakajima, Y. Honda, O. Kitao, H. Nakai, T. Vreven, J. A. Montgomery, Jr., J. E. Peralta, F. Ogliaro, M. Bearpark, J. J. Heyd, E. Brothers, K. N. Kudin, V. N. Staroverov, R. Kobayashi, J. Normand, K. Raghavachari, A. Rendell, J. C. Burant, S. S. Iyengar, J. Tomasi, M. Cossi, N. Rega, J. M. Millam, M. Klene, J. E. Knox, J. B. Cross, V. Bakken, C. Adamo, J. Jaramillo, R. Gomperts, R. E. Stratmann, O. Yazyev, A. J. Austin, R. Cammi, C. Pomelli, J. W. Ochterski, R. L. Martin, K. Morokuma, V. G. Zakrzewski, G. A. Voth, P. Salvador, J. J. Dannenberg, S. Dapprich, A. D. Daniels, Ö. Farkas, J. B. Foresman, J. V Ortiz, J. Cioslowski, D. J. Fox, *Gaussian 16*, *Revision C.01*, Gaussian, Inc., Wallingford CT, **2016**.

[18] S. Kundu , S. Sinhababu , V. Chandrasekhar , H. W. Roesky , Chem. Sci. 2019, 10, 4727–4741. 31160949 10.1039/c9sc01351b PMC6510188

[19] While the higher spin density at beryllium in **IV** may seem in contradiction to its lower hyperfine coupling constant to ^9^Be, the latter was merely computed and not derived from the experimental EPR spectrum of **IV**, which showed a broad featureless signal.

[20] Multiple attempts to obtain elemental analyses failed as [(CAAC)(CAACH)Be]^.^ already decomposed visibly, losing its red color, upon contact with the aluminum foil, in which the air-sensitive samples are encased.

[21] The disordered parts were refined using free variables and without applying any bond length or angle restraints.

[22] G. te Velde , F. M. Bickelhaupt , E. J. Baerends , C. Fonseca Guerra , S. J. A. van Gisbergen , J. G. Snijders , T. Ziegler , J. Comput. Chem. 2001, 22, 931–967.

[23a] M. Hermann , G. Frenking , Chem. Eur. J. 2017, 23, 3347–3356; 28004870 10.1002/chem.201604801

[23b] L. T. Scharf , D. M. Andrada , G. Frenking , V. H. Gessner , Chem. Eur. J. 2017, 23, 4422–4434; 28121371 10.1002/chem.201605997 PMC5396261

[23c] D. M. Andrada , J. L. Casals-Sainz , Á. Martín Pendás , G. Frenking , Chem. Eur. J. 2018, 24, 9083–9089; 29578617 10.1002/chem.201800680

[23d] A. Hermann , F. Fantuzzi , M. Arrowsmith , T. Zorn , I. Krummenacher , B. Ritschel , K. Radacki , B. Engels , H. Braunschweig , Angew. Chem. Int. Ed. 2020, 59, 15717–15725; 10.1002/anie.202006131 PMC7497145 32449598

[23e] C. Brunecker , J. H. Müssig , M. Arrowsmith , F. Fantuzzi , A. Stoy , J. Böhnke , A. Hofmann , R. Bertermann , B. Engels , H. Braunschweig , Chem. Eur. J. 2020, 26, 8518–8523. 32196775 10.1002/chem.202001168 PMC7384048

[24a] J. Böhnke , H. Braunschweig , W. C. Ewing , C. Hörl , T. Kramer , I. Krummenacher , J. Mies , A. Vargas , Angew. Chem. Int. Ed. 2014, 53, 9082–9085; 10.1002/anie.201403888 24864006

[24b] J.-S. Huang , W.-H. Lee , C.-T. Shen , Y.-F. Lin , Y.-H. Liu , S.-M. Peng , C.-W. Chiu , Inorg. Chem. 2016, 55, 12427–12434; 27934439 10.1021/acs.inorgchem.6b02336

[24c] J. Böhnke , T. Dellermann , M. A. Celik , I. Krummenacher , R. D. Dewhurst , S. Demeshko , W. C. Ewing , K. Hammond , M. Heß , E. Bill , E. Welz , M. I. S. Röhr , R. Mitrić , B. Engels , F. Meyer , H. Braunschweig , Nat. Commun. 2018, 9, 1197; 29567960 10.1038/s41467-018-02998-3 PMC5864745

[24d] E. Welz , J. Böhnke , R. D. Dewhurst , H. Braunschweig , B. Engels , J. Am. Chem. Soc. 2018, 140, 12580–12591; 30180566 10.1021/jacs.8b07644

[24e] C. Saalfrank , F. Fantuzzi , T. Kupfer , B. Ritschel , K. Hammond , I. Krummenacher , R. Bertermann , R. Wirthensohn , M. Finze , P. Schmid , V. Engel , B. Engels , H. Braunschweig , Angew. Chem. Int. Ed. 2020, 59, 19338–19343; 10.1002/anie.202008206 PMC7589216 32662218

[24f] P. Schmid , F. Fantuzzi , J. Klopf , N. B. Schröder , R. D. Dewhurst , H. Braunschweig , V. Engel , B. Engels , Chem. Eur. J. 2021, 27, 5160–5170; 33225473 10.1002/chem.202004619 PMC8048672

[24g] L. Wu , R. D. Dewhurst , H. Braunschweig , Z. Lin , Organometallics 2021, 40, 766–775.

[25] Deposition Numbers 2091667 ((CAAC)(CAACH)BeBr), 2091668 ((CAAC)BeBr_2_), 2091669 ((CAAC)(CAACH)BeCl) and 2091670 ([(CAAC)(CAACH)Be]^.^) contain the supplementary crystallographic data for this paper. These data are provided free of charge by the joint Cambridge Crystallographic Data Centre and Fachinformationszentrum Karlsruhe Access Structures service www.ccdc.cam.ac.uk/structures.

