# Supplementary material for: A Neutral Beryllium(I) Radical
Source: Angew Chem Int Ed Engl. 2021 Aug 24;60(38):20776–80. doi: 10.1002/anie.202108405 (PMC8518760; doi:10.1002/anie.202108405)
Supplement: Supplementary file 1 — Supporting Information [file ANIE-60-20776-s001.pdf]

Angewandte Chemie
Eine Zeitschrift der Gesellschaft Deutscher Chemiker

Supporting Information

A Neutral Beryllium(I) Radical

*Corinna Czernetzki, Merle Arrowsmith, Felipe Fantuzzi, Annalena Gärtner, Tobias Tröster, Ivo Krummenacher, Fabian Schorr, and Holger Braunschweig**

anie_202108405_sm_miscellaneous_information.pdf

Supporting Information

Contents

Methods and materials 2

Synthetic procedures 3

NMR spectra of isolated compounds 7

IR spectra 16

EPR spectroscopy 18

Cyclic voltammetry 19

UV-vis spectra 20

X-ray crystallographic details 22

Computational Details 27

Cartesian Coordinates 29

References 44

Methods and materials

All manipulations were performed either under an atmosphere of dry argon or *in vacuo* using standard Schlenk line or glovebox techniques. Deuterated solvents were dried over molecular sieves and degassed by three freeze-pump-thaw cycles prior to use. All other solvents were distilled and degassed from appropriate drying agents. Both deuterated and non-deuterated solvents were stored under argon over activated 4 Å molecular sieves. NMR spectra were acquired either on a Bruker Avance 500 (operating at 500 MHz for ^{1}H and 125 MHz for ^{13}C). or a Bruker Avance 400 NMR (operating at 400 MHz for ^{1}H, 56 MHz for ^{9}Be and 100 MHz for ^{13}C) spectrometer. Chemical shifts (δ) are yield in ppm and internally referenced to the carbon nuclei ($^{13}C\{^{1}H\}$) or residual protons (^{1}H) of the solventSolid-state IR spectra were recorded on a Bruker FT-IR spectrometer ALPHA II inside a glovebox. UV-vis spectra were acquired on a METTLER TOLEDO UV-vis-Excellence UV5 spectrophotometer inside a glovebox. Microanalyses (C, H, N, S) were performed on an Elementar vario MICRO cube elemental analyzer. Electron paramagnetic resonance (EPR) measurements at the X band (9.38 GHz) were carried out at room temperature using a Bruker ELEXSYS E580 EPR spectrometer equipped with an Oxford Instruments helium cryostat (ESR 900) and a MercuryiTC temperature controller. *Note: both elemental analyses and HRMS were carried out for all new compounds but in some cases these decomposed too rapidly and only one type of analysis was possible.*

L-Selectride and beryllium metal were purchased from various chemicals companies, transferred into a glovebox and used as received. $BeCl_2$,[1] $(Et_2O)BeBr_2$,[2] $CAAC^{Me}$ (1-(2,6-di*iso*propylphenyl)-3,3,5,5-tetramethyl-pyrrolidin-2-ylidene)[3] and $(CAAC)BeCl_2$[4] were synthesized following literature procedures.

Process of silanization: 0.5 mL hexamethyldisilazane (HMDS) was heated in a vial with a heat gun until the HMDS evaporated.

Synthetic procedures

(CAAC)(CAACH)BeCl

A suspension of CAAC (156 mg, 0.547 mmol) and (CAAC)$BeCl_2$ (200 mg, 0.547 mmol) in 10 mL of toluene was cooled to –78 °C and L-Selectride (0.550 mL, 1.00 M in THF, 0.547 mmol) was added dropwise. The reaction mixture was warmed to room temperature and left to stir overnight. After removal of volatiles *in vacuo*, the residual orange solid was washed with pentane until the washing phases became colorless, and then extracted with benzene. After removal of the solvent *in vacuo* (CAAC)(CAACH)BeCl was isolated as an orange solid (275 mg, 0.446 mmol, 82% yield). ^{1}H NMR (500.1 MHz, C_6D_6): δ = 7.36 (dd, ^3J = 7.6 Hz, ^4J = 1.8 Hz, 1H, *m*-C*H*$_{Ar}$), 7.23 (t, ^3J = 7.6 Hz, 1H, *p*-C*H*$_{Ar}$), 7.08 (m, 2H, C*H*$_{Ar}$), 7.15 (s, 1H, C*H*$_{Ar}$), 6.91 (m, 1H, C*H*$_{Ar}$), 4.88 (sept., ^3J = 6.6 Hz, 1H, C*H*(CH_3)$_2$), 3.56 (sept., ^3J = 6.7 Hz, 1H, C*H*(CH_3)$_2$), 3.24 (sept., ^3J = 6.5 Hz, 1H, C*H*(CH_3)$_2$), 2.97 (s, 1H, BeC*H*), 2.4 (sept., ^3J = 6.8 Hz, 1H, C*H*(CH_3)$_2$), 2.14 (d, ^2J = 11.8 Hz, 1H, C*H*$_2$), 1.90 (s, 3H, C(C*H*$_3$)$_2$), 1.85 (d, ^2J = 11.7 Hz, 1H, C*H*$_2$), 1.76 (d, ^3J = 6.7 Hz, 3H, CH(C*H*$_3$)$_2$), 1.60 (d, ^3J = 6.5 Hz, 3H, CH(C*H*$_3$)$_2$), 1.45 (d, ^3J = 6.6 Hz, 3H, CH(C*H*$_3$)$_2$), 1.40 (s, 3H, C(C*H*$_3$)$_2$), 1.38 (d, ^3J = 6.6 Hz, 3H, CH(C*H*$_3$)$_2$), 1.31-1.33 (m, 6H, C(C*H*$_3$)$_2$ + CH(C*H*$_3$)$_2$), 1.28 (d, ^3J = 6.8 Hz, 3H, CH(C*H*$_3$)$_2$), 1.24 (s, 3H, C(C*H*$_3$)$_2$), 1.18-1.15 (m, 2H, C*H*$_2$), 1.09 (s, 3H, C(C*H*$_3$)$_2$), 1.03 (d, ^3J = 6.5 Hz, 3H, CH(C*H*$_3$)$_2$), 0.98 (d, ^3J = 6.8 Hz, 3H, CH(C*H*$_3$)$_2$), 0.79 (s, 3H, C(C*H*$_3$)$_2$), 0.69 (s, 3H, C(C*H*$_3$)$_2$), 0.59 (s, 3H, C(C*H*$_3$)$_2$) ppm. ^{13}C{^{1}H} NMR (125.8 MHz, C_6D_6): δ = 250.7 (*C*$_{carbene}$), 153.3 (*o*-*C*$_{Ar}$), 151.3 (*o*-*C*$_{Ar}$), 147.4 (N*C*$_{Ar}$), 145.8 (*o*-*C*$_{Ar}$), 145.7 (*o*-*C*$_{Ar}$), 135.5 (N*C*$_{Ar}$), 129.9 (*C*H$_{Ar}$), 126.4 (*C*H$_{Ar}$), 125.3 (*C*H$_{Ar}$), 125.2 (*p*-*C*H$_{Ar}$), 125.0 (*C*H$_{Ar}$), 124.2 (*m*-*C*H$_{Ar}$), 80.7 (N*C*(CH_3)$_2$), 71.2 (Be*C*H), 63.9 (N*C*(CH_3)$_2$), 62.5 (*C*H$_2$), 54.9 (*C*(CH_3)$_2$), 51.8 (*C*H$_2$), 41.6 (*C*(CH_3)$_2$), 34.2 (C(*C*H$_3$)$_2$), 33.5 (C(*C*H$_3$)$_2$), 33.4 (C(*C*H$_3$)$_2$), 30.6 (C(*C*H$_3$)$_2$), 30.3 (C(*C*H$_3$)$_2$), 29.5 (C(*C*H$_3$)$_2$), 29.4 (*C*H(CH_3)$_2$), 29.2 (C(*C*H$_3$)$_2$), 29.0 (*C*H(CH_3)$_2$), 28.8 (*C*H(CH_3)$_2$), 28.8 (CH(*C*H$_3$)$_2$), 27.8 (CH(*C*H$_3$)$_2$), 27.7 (CH(*C*H$_3$)$_2$), 27.4 (*C*H(CH_3)$_2$), 26.2 (C(*C*H$_3$)$_2$), 25.9 (CH(*C*H$_3$)$_2$), 25.9 (CH(*C*H$_3$)$_2$), 25.8 (CH(*C*H$_3$)$_2$), 25.7 (CH(*C*H$_3$)$_2$), 23.8 (CH(*C*H$_3$)$_2$) ppm. ^{9}Be NMR (56 MHz, C_6D_6): δ = 19 ppm. UV-vis (Et_2O): λ_1 = 244 nm, λ_2 = 325 nm, λ_3 = 404 nm. Elemental analysis (%) calcd. for $C_{40}H_{63}BeClN_2$ [616.42 g mol^{-1}]: C 77.94, H 10.30, N 4.54; found: C 77.78, H 10.31, N 4.42.

(CAAC)BeBr$_2$

BeBr$_2$(OEt)$_2$ (300 mg, 0.946 mmol, 1.00 equiv.) was added to a white suspension of CAAC (297 mg, 1.04 mmol, 1.10 equiv.) in 6 mL of benzene. After 5 h the solvent was removed *in vacuo* and the resulting residue was washed with hexane (3 × 2 mL) and benzene (3 × 2 mL). Drying *in vacuo* afforded (CAAC)BeBr$_2$ as a colorless solid (377 mg, 0.831 mmol, 88%). ^{1}H NMR (500.1 MHz, CDCl$_3$): = 7.34 (t, 1H, ^3J = 7.8 Hz, *p*-C*H*$_{Ar}$), 7.19 (d, 2H, ^3J = 7.8 Hz, 2H, *m*-C*H*$_{Ar}$), 2.68 (sept., 2H, ^3J = 6.6 Hz, C*H*(CH$_3$)$_2$), 2.00 (s, 2H, C*H*$_2$), 1.61 (s, 6H, C(C*H*$_3$)$_2$), 1.34 (s, 6H, C(C*H*$_3$)$_2$), 1.30 (d, ^3J = 6.6 Hz, 6H, CH(C*H*$_3$)$_2$), 1.22 (d, ^3J = 6.6 Hz, 6H, CH(C*H*$_3$)$_2$) ppm. ^{13}C{^{1}H} NMR (125.8 MHz, CDCl$_3$): δ = 239.0 (*C*$_{carbene}$), 145.4 (*o*-*C*$_{Ar}$), 132.8 (N*C*$_{Ar}$), 130.3 (*p*-*C*H$_{Ar}$), 125.7 (*m*-*C*H$_{Ar}$), 83.1 (N*C*(CH$_3$)$_2$), 54.7 (*C*(CH$_3$)$_2$), 51.0 (*C*H$_2$), 29.5 (C(*C*H$_3$)$_2$), 29.2 (C(*C*H$_3$)$_2$), 28.8 (*C*H(CH$_3$)$_2$), 27.6 (CH(*C*H$_3$)$_2$), 25.1 (CH(*C*H$_3$)$_2$) ppm. ^{9}Be NMR (56 MHz, CDCl$_3$): δ = 14 ppm. Elemental analysis (%) calcd. for C$_{40}$H$_{63}$BeBrN$_2$ [454.29 g mol^{-1}]: C 52.88, H 6.88, N 3.08; found.: C 52.15, H 6.89, N 3.04.

(CAAC)(CAACH)BeBr

A solution of CAAC (316 mg, 0.759 mmol, 1.00 equiv.) and (CAAC)BeBr$_2$ (300 mg, 0.660 mmol, 1.00 equiv.) in 15 mL of toluene was cooled to –78 °C and L-Selectride (0.66 mL, 1.00 M in THF, 0.660 mmol, 1.00 equiv.) was added dropwise. The reaction was warmed to room temperature and stirred overnight. After removal of volatiles *in vacuo*, the residual orange solid was washed with pentane until the washing phases became colorless, and then extracted with benzene. After removal of the solvent *in vacuo* (CAAC)(CAACH)BeBr was isolated as an orange solid (385 mg, 0.583 mmol, 88% yield). ^{1}H NMR (500.1 MHz, C$_6$D$_6$): δ = 7.35 (dd, ^3J = 7.5 Hz, ^4J = 1.8 Hz, 1H, *m*-C*H*$_{Ar}$), 7.23 (t, ^3J = 7.5 Hz, 1H, *p*-C*H*$_{Ar}$), 7.14 (m, 1H, *m*-C*H*$_{Ar}$), 7.08 (s, 2H, C*H*$_{Ar}$), 6.91 (m, 1H, C*H*$_{Ar}$), 4.94 (sept., ^3J = 6.7 Hz, 1H, C*H*(CH$_3$)$_2$), 3.53 (sept., ^3J = 6.7 Hz, 1H, C*H*(CH$_3$)$_2$), 3.37 (sept., ^3J = 6.5 Hz, 1H, C*H*(CH$_3$)$_2$), 3.03 (s, 1H, BeC*H*), 2.36 (sept., ^3J = 6.8 Hz, 1H, C*H*(CH$_3$)$_2$), 2.16 (d, ^2J = 11.8 Hz, 1H, C*H*$_2$), 1.98 (s, 3H, C(C*H*$_3$)$_2$), 1.85 (d, ^2J = 11.8 Hz, 1H, C*H*$_2$), 1.75 (d, ^3J = 6.7 Hz, 3H, CH(C*H*$_3$)$_2$), 1.61 (d, ^3J = 6.5 Hz, 3H, CH(C*H*$_3$)$_2$), 1.54 (s, 3H, C(C*H*$_3$)$_2$), 1.45 (d, ^3J = 6.7 Hz, 3H, CH(C*H*$_3$)$_2$), 1.38 (d, ^3J = 6.7 Hz, 3H, CH(C*H*$_3$)$_2$), 1.32 (d, ^3J = 6.7 Hz, 3H, CH(C*H*$_3$)$_2$), 1.28 – 1.25 (m, 7H, C(C*H*$_3$)$_2$ + CH(C*H*$_3$)$_2$ + C*H*$_2$ detected by HSQC), 1.22 (s, 3H, C(C*H*$_3$)$_2$), 1.18 (s, 3H, C(C*H*$_3$)$_2$), 1.14 (d, ^2J = 12.8 Hz, 1H, C*H*$_2$), 1.02 (d, ^3J = 6.5 Hz, 3H, CH(C*H*$_3$)$_2$), 0.97 (d, ^3J = 6.8 Hz, 3H, CH(C*H*$_3$)$_2$), 0.86 (s, 3H, C(C*H*$_3$)$_2$), 0.69 (s, 3H, C(C*H*$_3$)$_2$), 0.59 (s, 3H, C(C*H*$_3$)$_2$) ppm. ^{13}C{^{1}H} NMR (125.8 MHz, C$_6$D$_6$): δ = 249.7 (*C*$_{carbene}$), 153.1 (*o*-*C*$_{Ar}$), 151.1 (*o*-*C*$_{Ar}$), 147.6 (N*C*$_{Ar}$), 146.0 (*o*-*C*$_{Ar}$), 145.6 (*o*-

C$_{Ar}$), 135.7 (N*C*$_{Ar}$), 130.0 (*C*H$_{Ar}$), 126.4 (*C*H$_{Ar}$), 125.3 (*C*H$_{Ar}$), 125.2 (*p*-*C*H$_{Ar}$), 125.0 (*m*-*C*H$_{Ar}$), 124.2 (*m*-*C*H$_{Ar}$), 80.5 (N*C*(CH$_3$)$_2$), 71.6 (*C*H, Be*C*H), 64.0 (N*C*(CH$_3$)$_2$), 62.2 (*C*H$_2$), 54.9 (*C*(CH$_3$)$_2$), 52.0 (*C*H$_2$), 41.6 (*C*(CH$_3$)$_2$), 34.8 (C(*C*H$_3$)$_2$), 33.1 (C(*C*H$_3$)$_2$), 33.3 (C(*C*H$_3$)$_2$), 30.5 (C(*C*H$_3$)$_2$), 30.5 (C(*C*H$_3$)$_2$), 30.4 (C(*C*H$_3$)$_2$), 29.3 (*C*H(CH$_3$)$_2$), 29.2 (C(*C*H$_3$)$_2$), 28.9 (*C*H(CH$_3$)$_2$), 28.8 (*C*H(CH$_3$)$_2$), 28.6 (CH(*C*H$_3$)$_2$), 27.9 (CH(*C*H$_3$)$_2$), 27.9 (C(*C*H$_3$)$_2$), 27.5 (*C*H(CH$_3$)$_2$), 26.2 (C(*C*H$_3$)$_2$), 26.2 (CH(*C*H$_3$)$_2$), 25.9 (CH(*C*H$_3$)$_2$), 25.7 (CH(*C*H$_3$)$_2$), 25.0 (C(*C*H$_3$)$_2$), 23.7 (CH(*C*H$_3$)$_2$) ppm. ^{9}Be NMR (56 MHz, C_6D_6): $\delta = 20$ ppm. UV-vis (Et_2O): $\lambda_1 = 242$ nm, $\lambda_2 = 266$ nm, $\lambda_3 = 325$ nm, $\lambda_4 = 425$ nm. Elemental analysis (%) calcd. for $C_{40}H_{63}BeBrN_2$ [660.87 g mol^{-1}]: C 72.70, H 9.61, N 4.24; found.: C 71.99, H 9.43, N 4.16.

[(CAAC)(CAACH)Be]$^•$

In a silanized vial (CAAC)(CAACH)BeCl (100 mg, 0.162 mmol, 1.00 equiv.) was suspended in Et_2O and lithium sand (30.0 mg, 4.32 mmol, 26.0 equiv.) was added. The reaction mixture was stirred for 10 min until the color of the suspension changed from orange to brown. Residual lithium sand was filtered off and the filtrate was dried *in vacuo*. The solid residue was extracted with benzene and the resulting solution freeze-dried to yield [(CAAC)(CAACH)Be]$^•$ as a light brown powder (66.3 mg, 0.114 mmol, 70% yield). Orange single crystals were obtained from a concentrated benzene solution left undisturbed in a silanized glass vial or polyethylene plastic vial in the glovebox for one day. EPR (CW, X-band, benzene, rt): $g_{iso} = 2.003$; $a(^9Be) = 11.6$ MHz, $a(^{14}N) = 3.7$ MHz, $a(^1H) = 6.3$ MHz. UV-vis (Et_2O): $\lambda_{max} = 350$ nm. *Note: multiple attempts to obtain elemental analyses failed as [(CAAC)(CAACH)Be]$^•$ already decomposed visibly, losing its orange color upon contact with the aluminum foil, in which the air-sensitive samples are encased.*

Failed attempts at synthesizing [(CAAC)(CAACH)Be]$^•$

A number of other reducing agents and reaction conditions were tried, albeit unsuccessfully, to obtain [(CAAC)(CAACH)Be]$^•$:

<u>From (CAAC)(CAACH)BeCl:</u>

- Sodium sand mixed with NaCl in diethyl ether: no reaction;
- Potassium chunks or KC_8 in diethyl ether or benzene: decomposition;

- Lithium chunks, lithium sand, sodium chunks, sodium sand in NaCl or potassium chunks in THF or dimethyl ether: decomposition;
- Lithium chunks in diethyl ether: successful reduction but problems with reproducibility and overreduction.

From (CAAC)(CAACH)BeBr:

- Lithium sand in diethyl ether or benzene: decomposition and isolation of [CAACH]Br.
- KC_8 in benzene: decomposition and isolation of [CAACH]Br.
- Decamethylcobaltocene in benzene: partial reduction to $[(CAAC)(CAACH)Be]^{\bullet}$ but still some precursor left even when using an excess of decamethylcobaltocene.

NMR spectra of isolated compounds

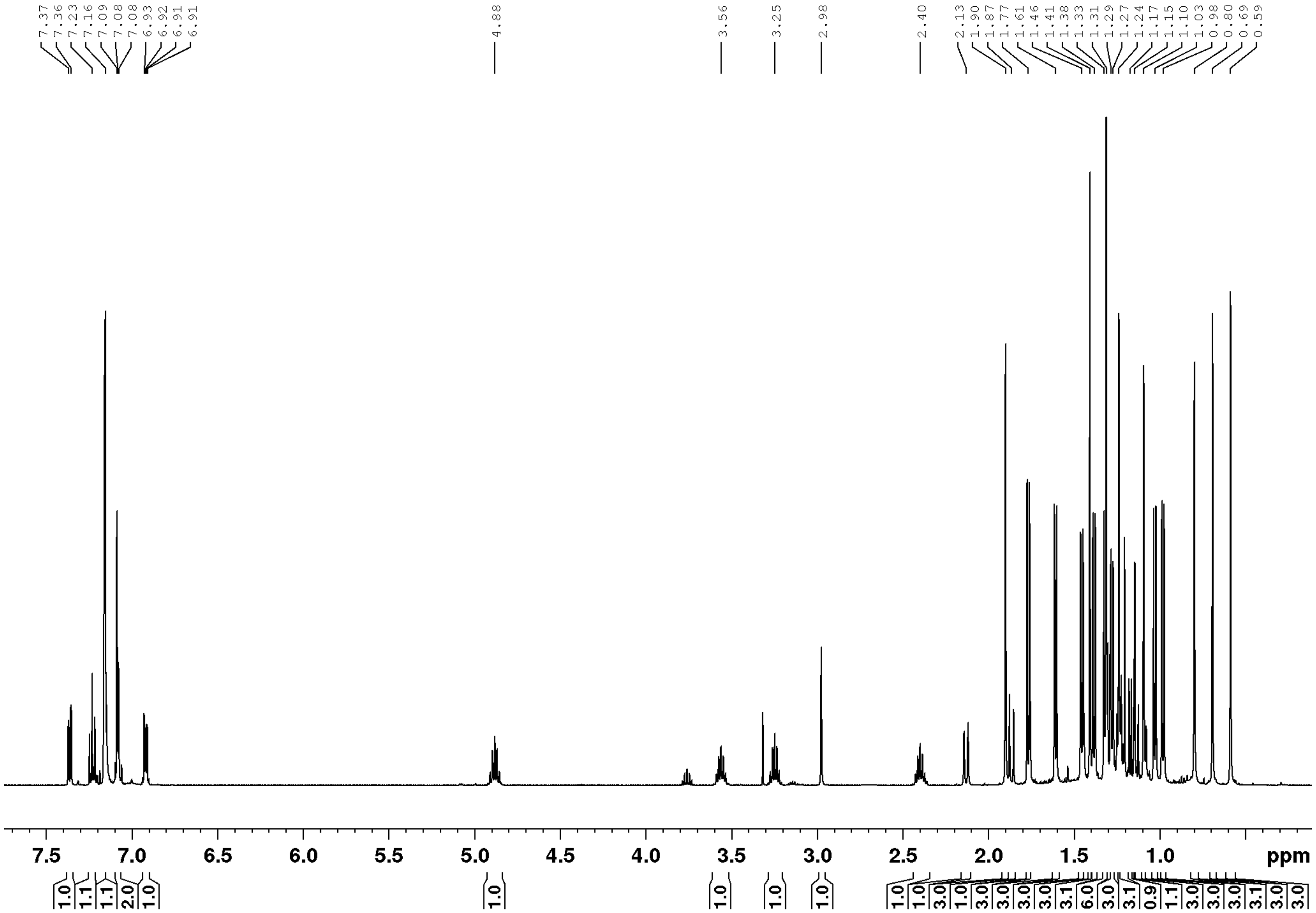

Figure S1. ^{1}H NMR spectrum of (CAAC)(CAACH)BeCl in C_6D_6.

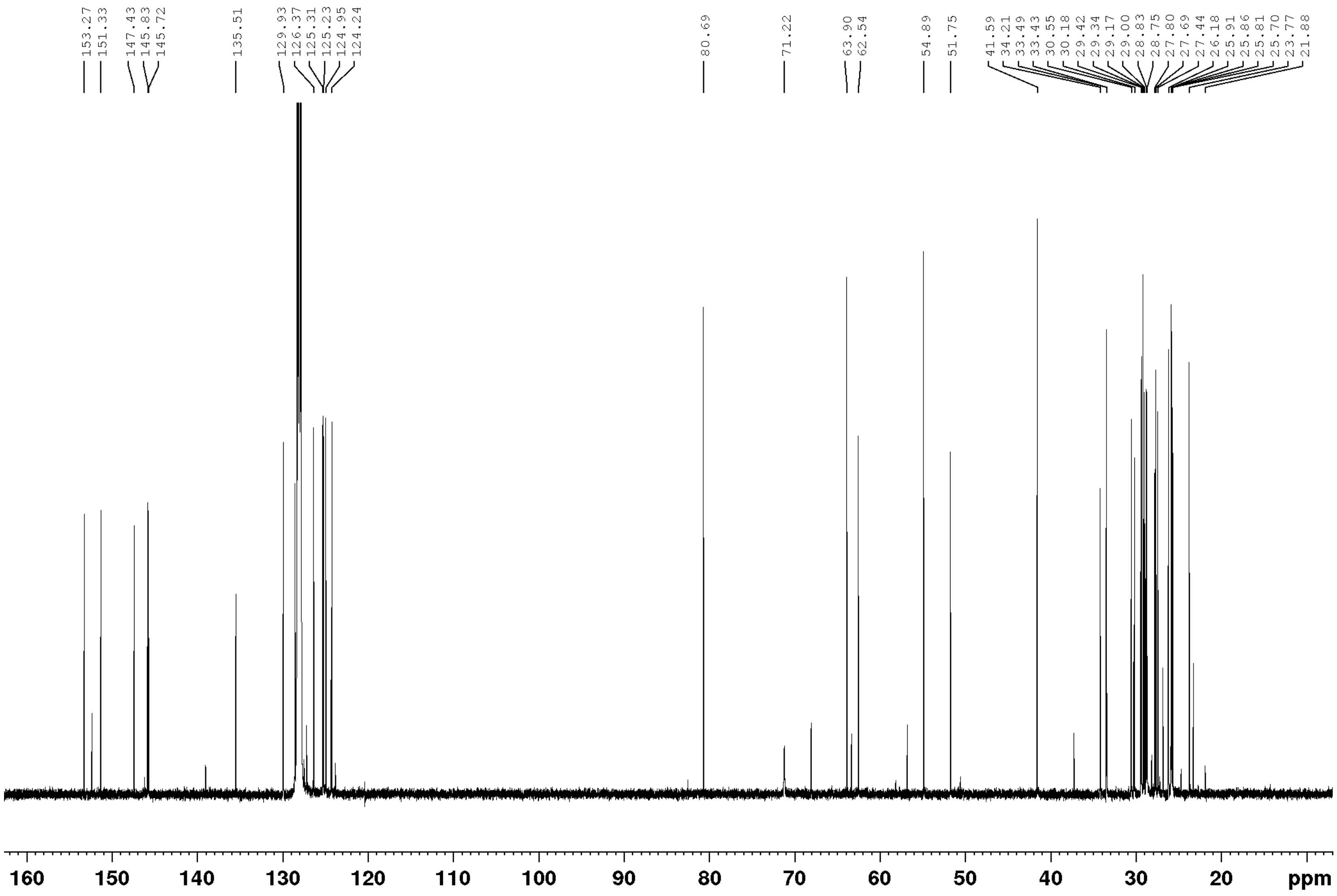

Figure S2. $^{13}C\{^{1}H\}$ NMR spectrum of (CAAC)(CAACH)BeCl in C_6D_6.

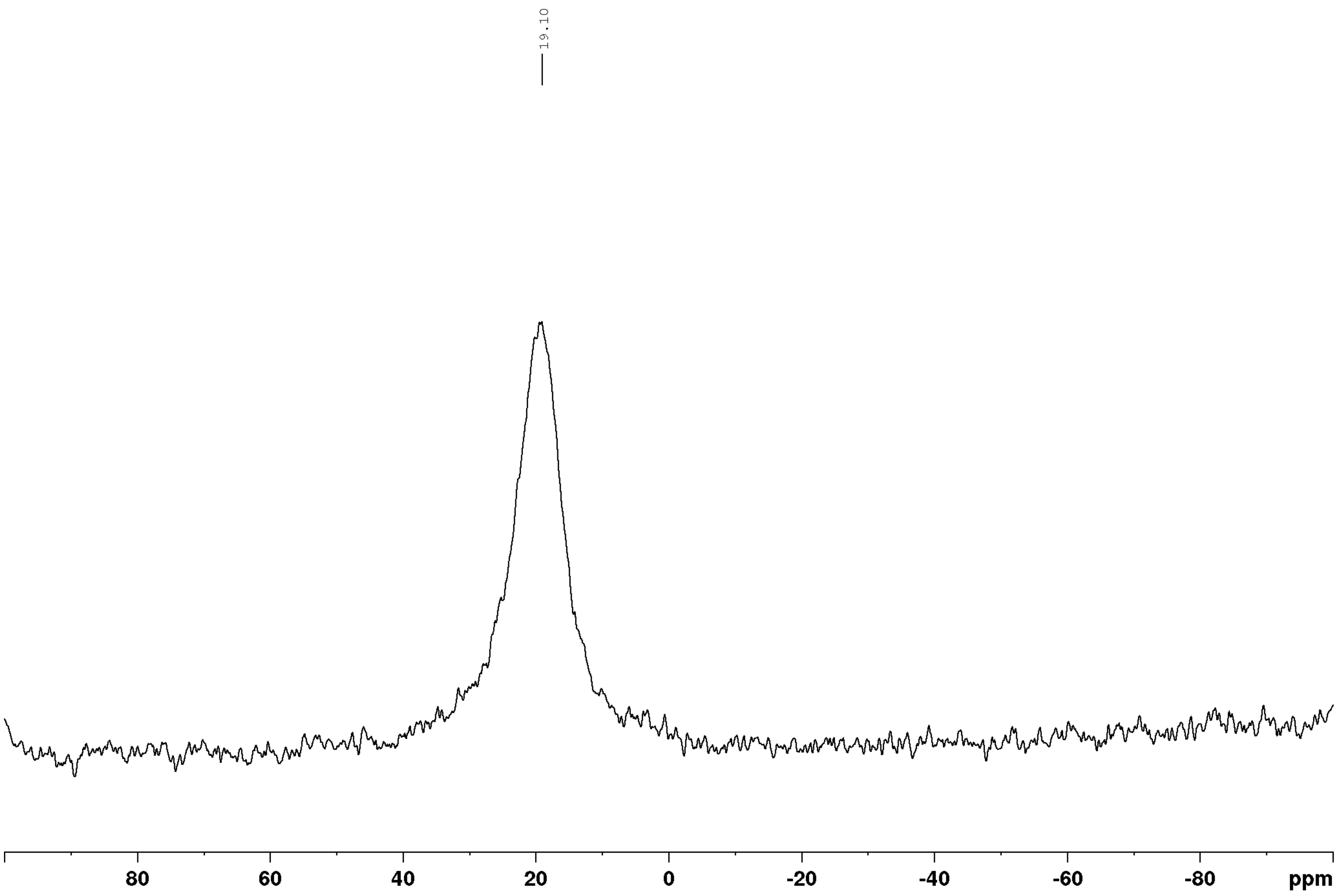

Figure S3. ^{9}Be NMR spectrum of (CAAC)(CAACH)BeCl in C_6D_6.

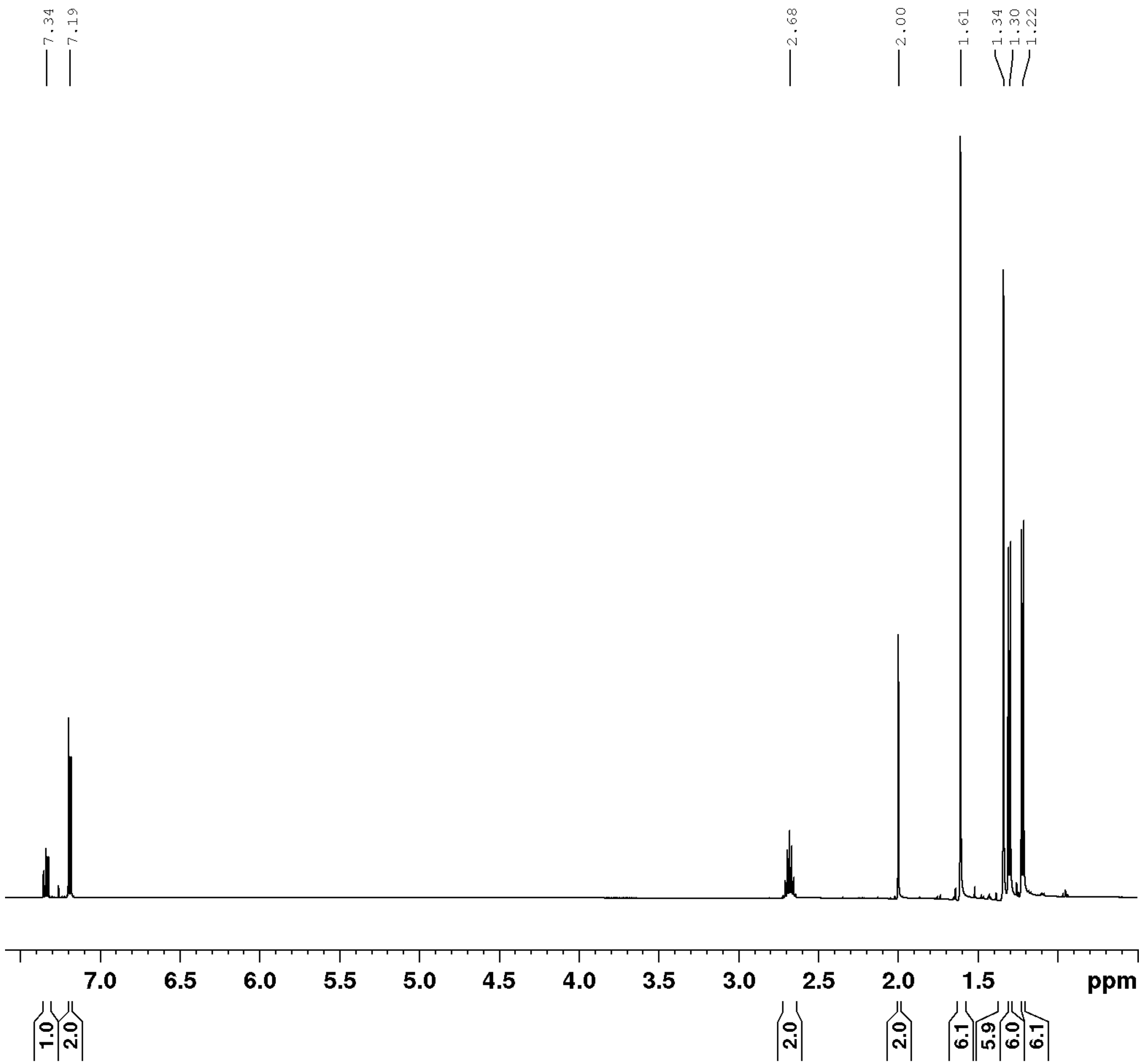

Figure S4. ^{1}H NMR spectrum of (CAAC)$BeBr_2$ in $CDCl_3$.

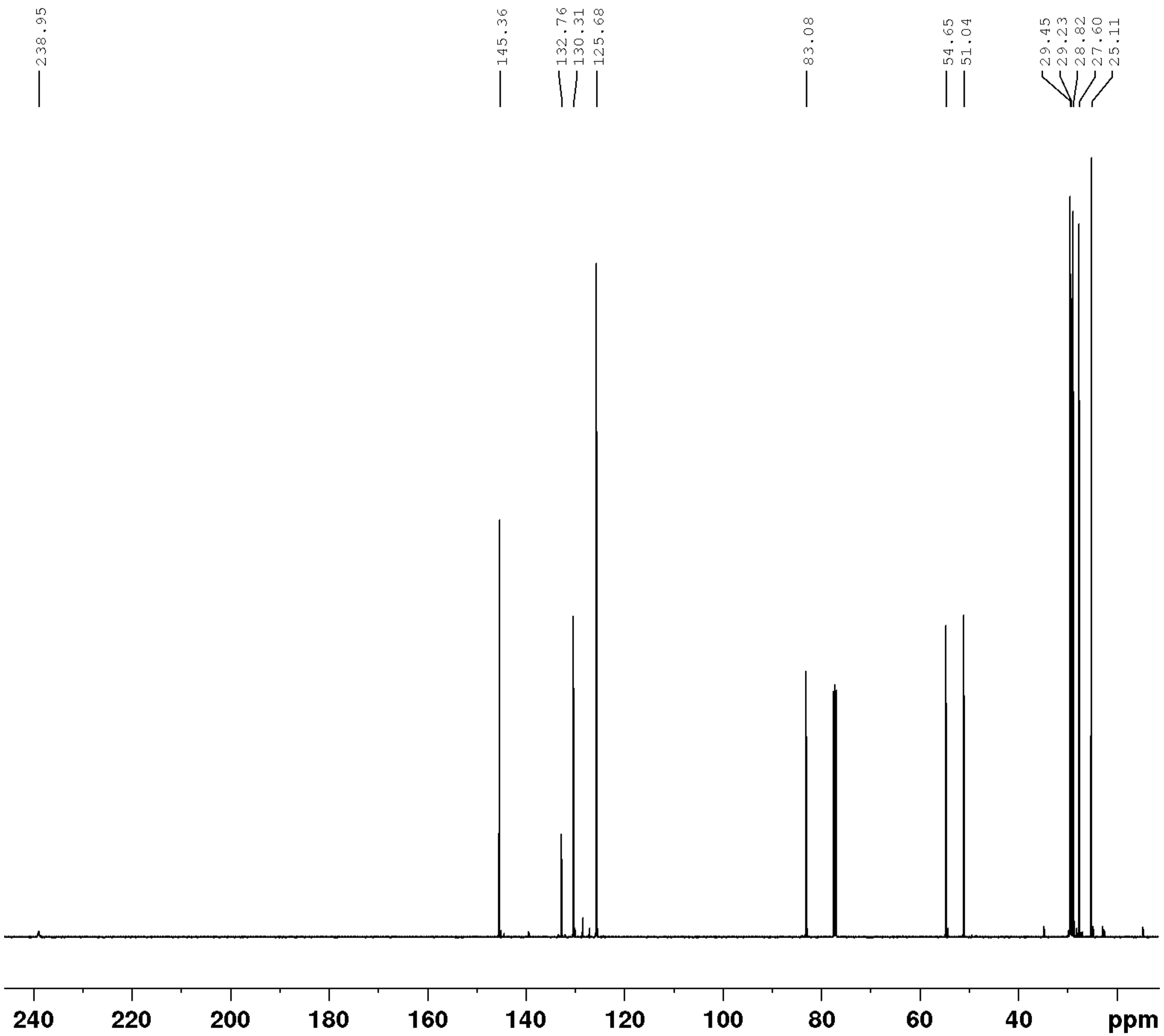

Figure S5. $^{13}C\{^{1}H\}$ NMR spectrum of (CAAC)$BeBr_2$ in $CDCl_3$.

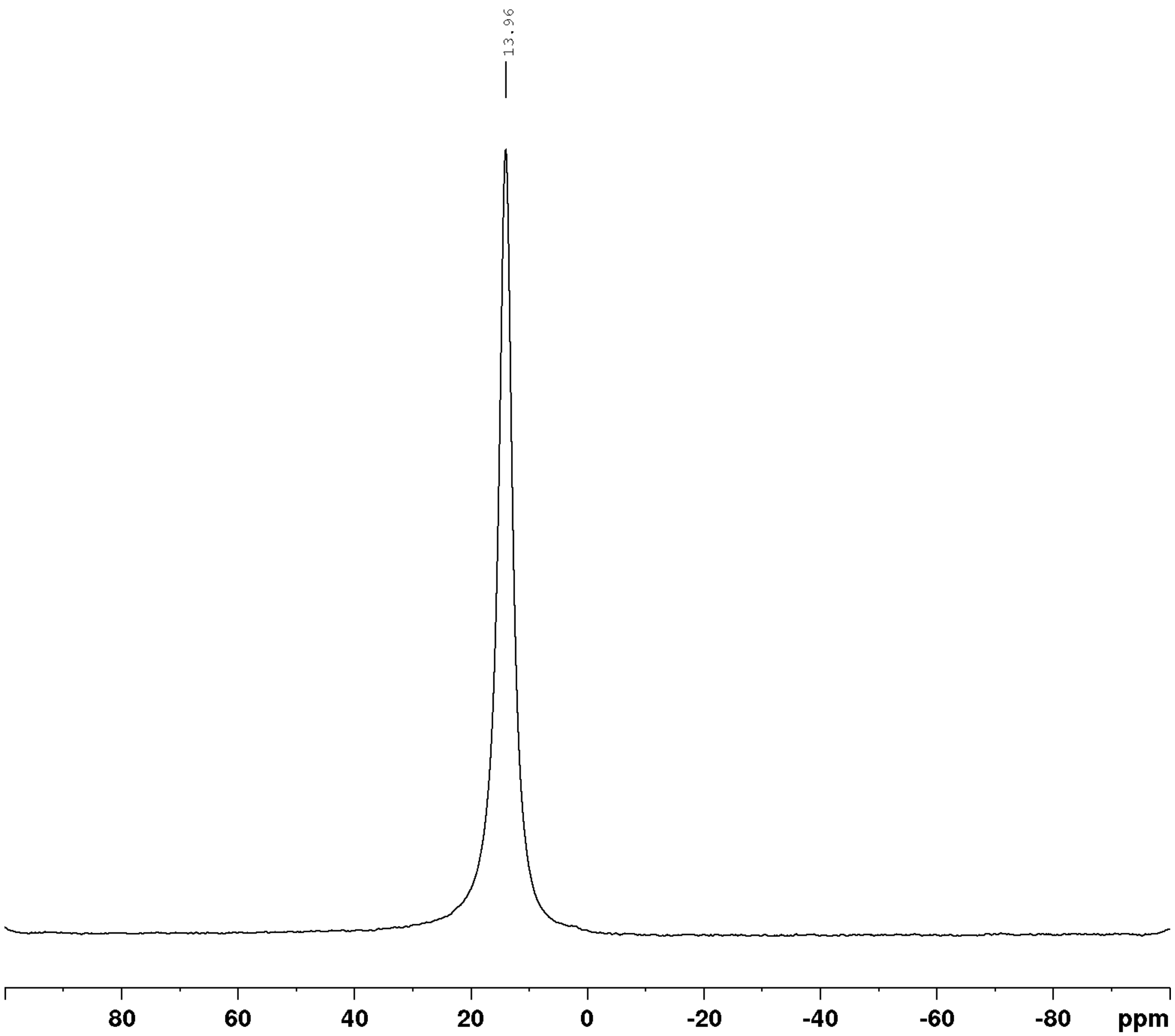

Figure S6. ^{9}Be NMR spectrum of (CAAC)$BeBr_2$ in $CDCl_3$.

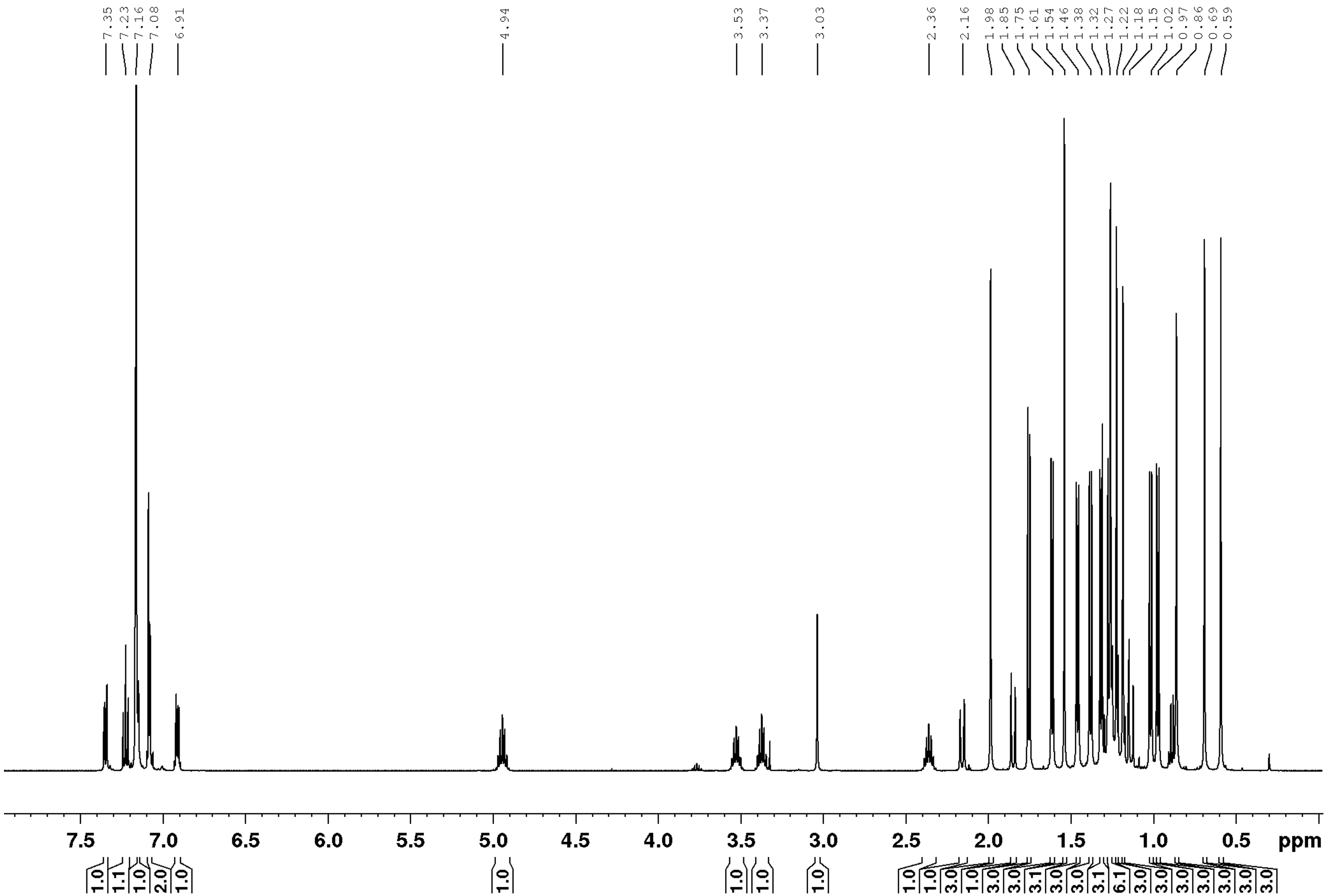

Figure S7. ^{1}H NMR spectrum of (CAAC)(CAACH)BeBr in C_6D_6.

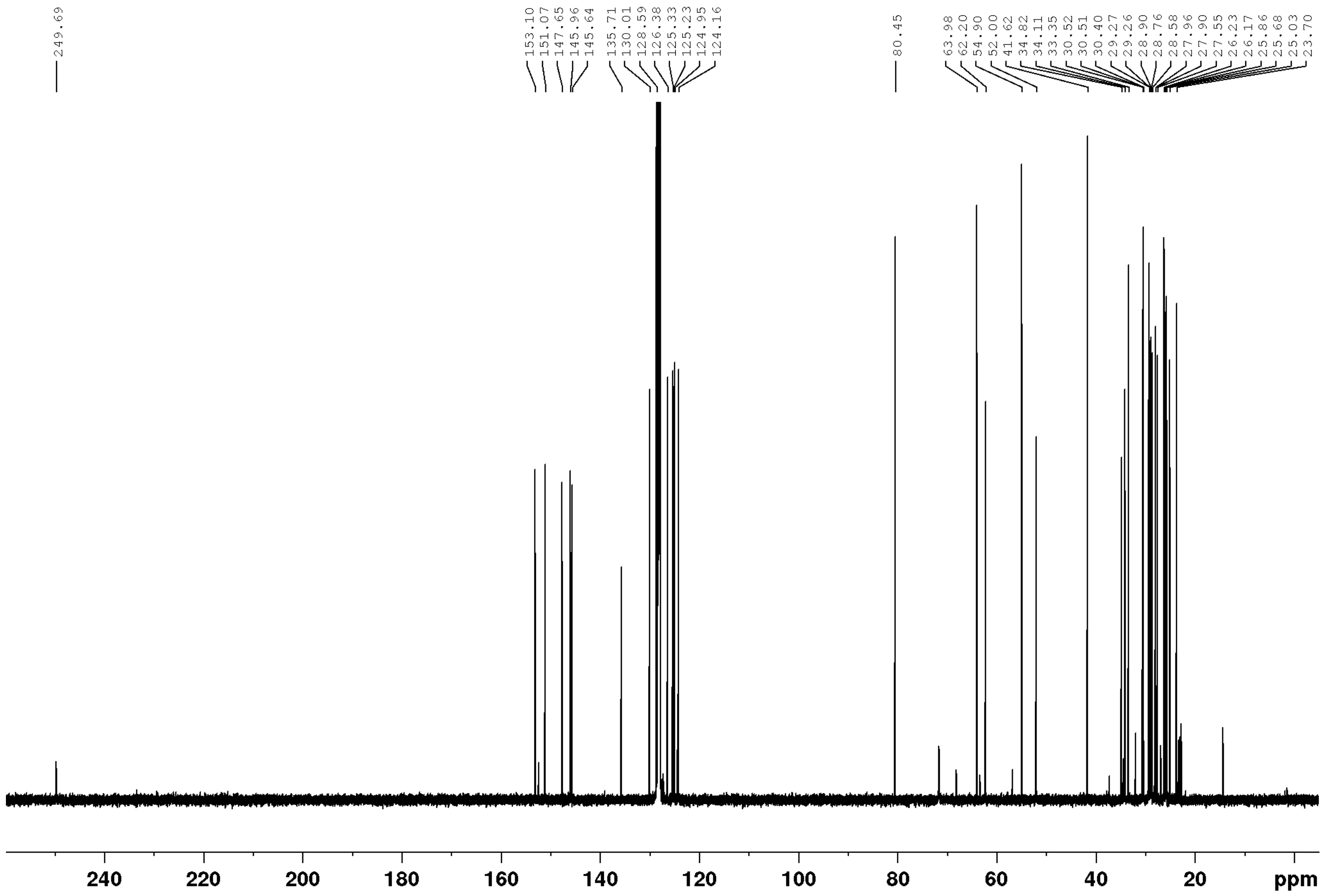

Figure S8. $^{13}C\{^1H\}$ NMR spectrum of (CAAC)(CAACH)BeBr in C_6D_6.

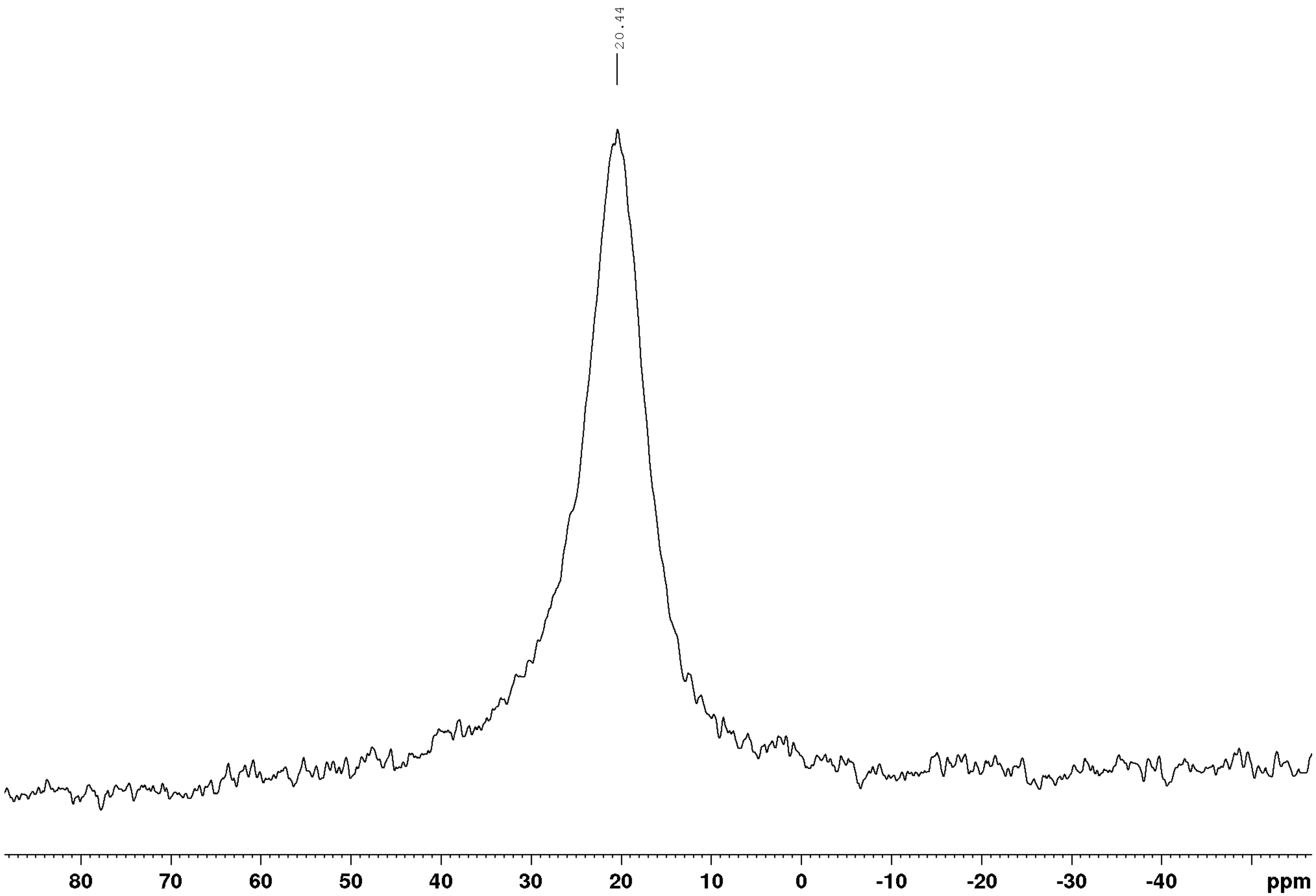

Figure S9. ^{9}Be NMR spectrum of (CAAC)(CAACH)BeBr in C_6D_6.

IR spectra

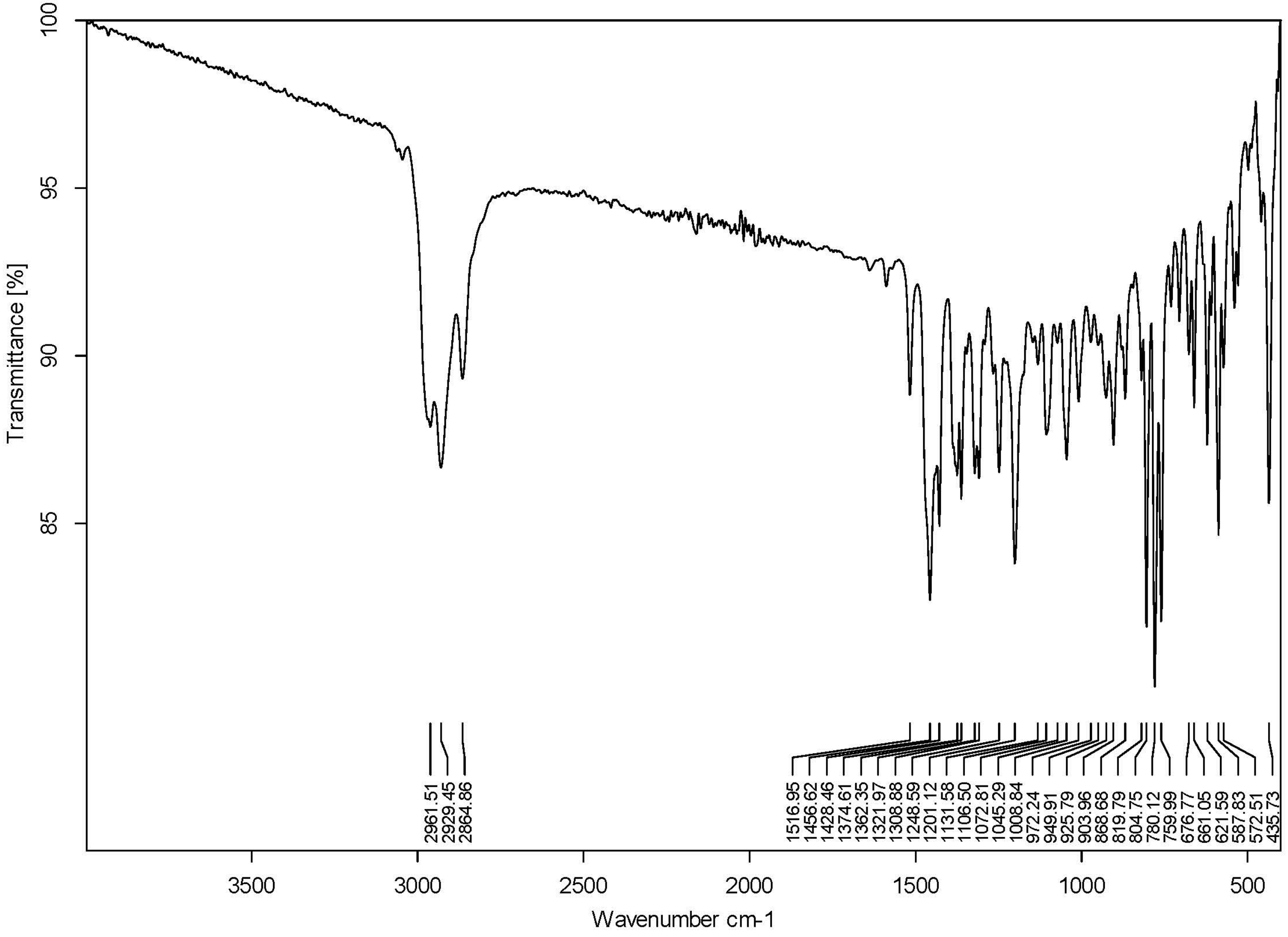

Figure S10. Solid-state IR spectrum of (CAAC)(CAACH)BeCl.

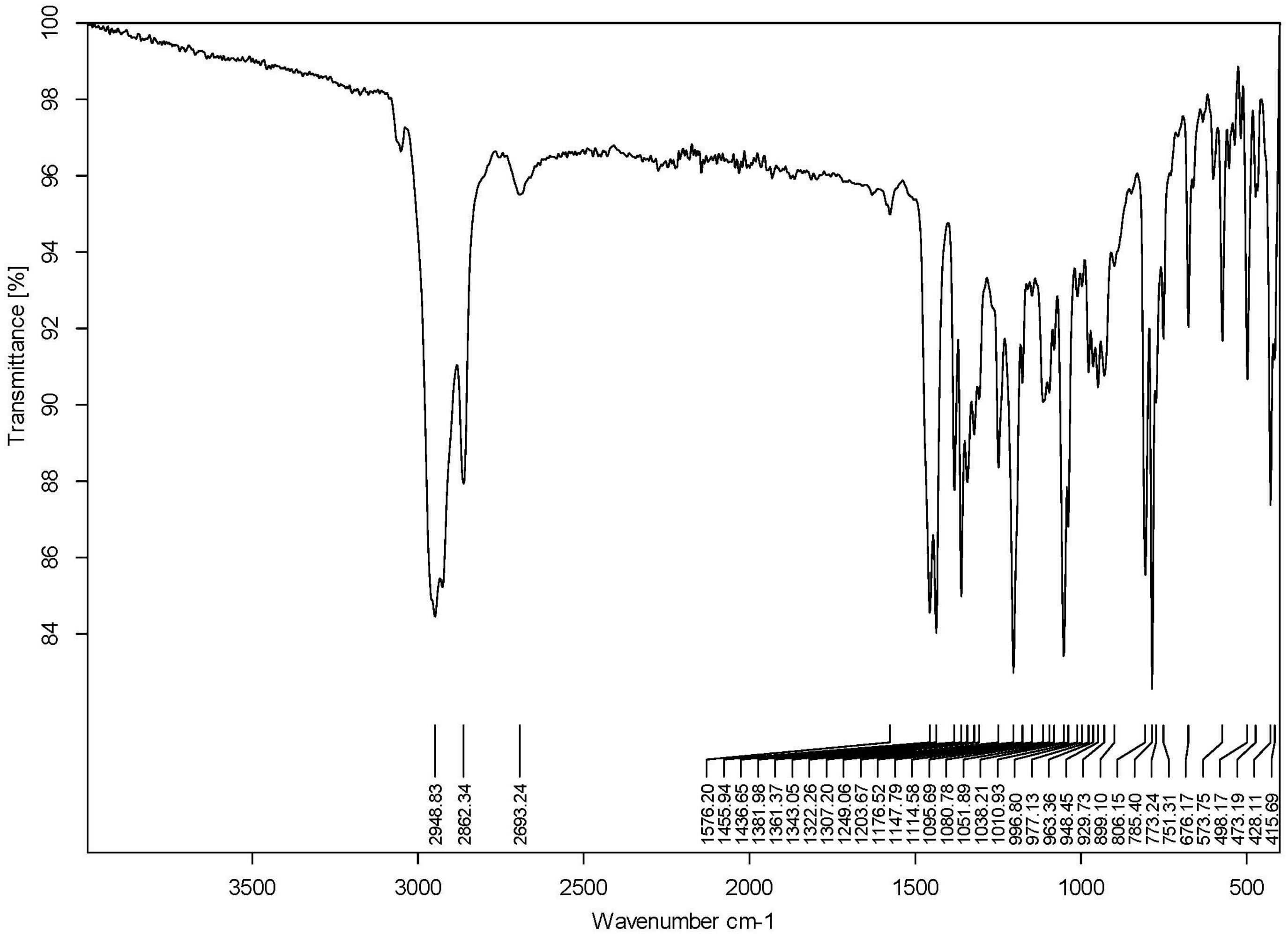

Figure S11. Solid-state IR spectrum of [(CAAC)(CAACH)Be]$^{\bullet}$. The absence of a Be–H stretching band (calculated to appear around 1800 cm^{-1}, see Figure S22) indicates the absence of the putative isomer [(CAAC)$_2$BeH]$^{\bullet}$.

EPR spectroscopy

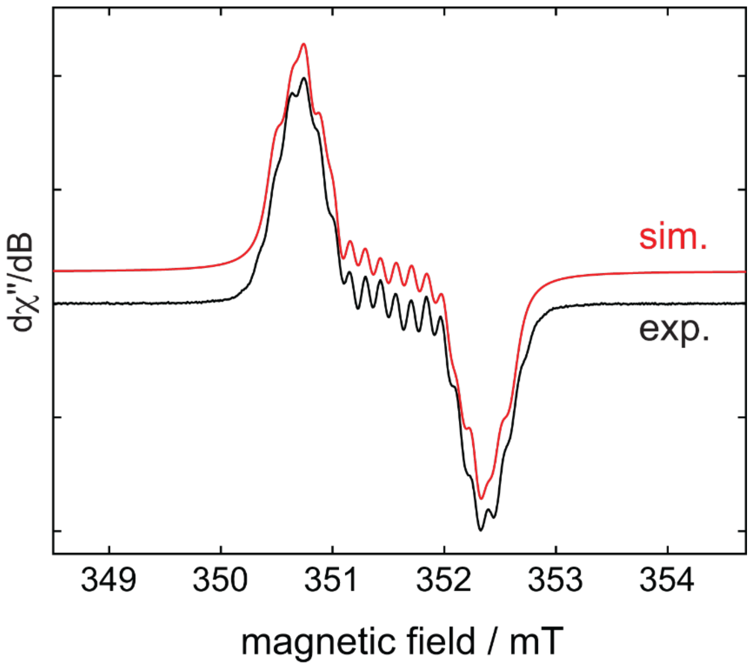

Figure S12. Experimental (black) and simulated (red) continuous-wave (CW) X-band EPR spectra of [(CAAC)(CAACH)Be]$^{\bullet}$ in benzene at room temperature. Experimental parameters: microwave frequency = 9.85 GHz; microwave power = 0 2 mW; modulation amplitude = 0.5 G; conversion time = 20 ms; modulation frequency = 100 kHz. The simulation parameters are g_{iso} = 2.003, $a(^{9}Be)$ = 11.6 MHz, $a(^{14}N)$ = 3.7 MHz, and $a(^{1}H)$ = 6.3 MHz.

Cyclic voltammetry

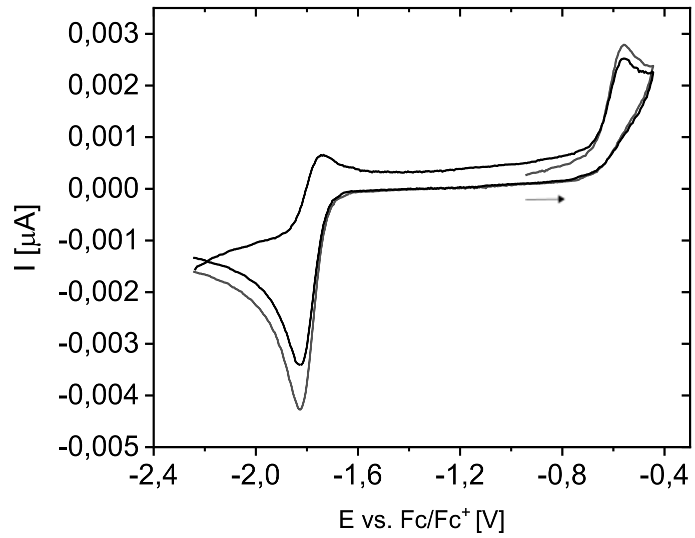

Figure S13. Cyclic voltammogram of (CAAC)(CAACH)BeCl in *o*-difluorobenzene with 0.1 M [*n*Bu_4N][PF_6] as the supporting electrolyte (relative to Fc/Fc^+ couple with a scan rate of 250 mV/s). Irreversible reduction: E_{pc} = –1.83 V.

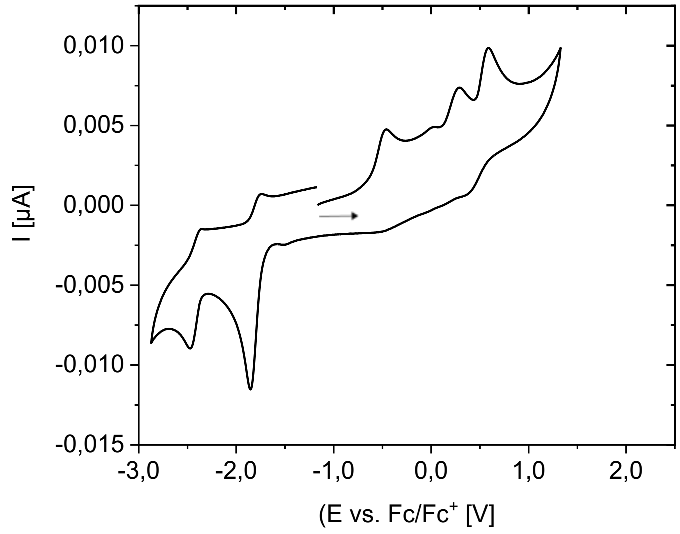

Figure S14. Cyclic voltammogram of (CAAC)(CAACH)BeBr in *o*-difluorobenzene with 0.1 M [*n*Bu_4N][PF_6] as the supporting electrolyte (relative to Fc/Fc^+ couple with a scan rate of 250 mV/s). Irreversible reductions: E_{pc} = –1.85 V and –2.46 V.

UV-vis spectra

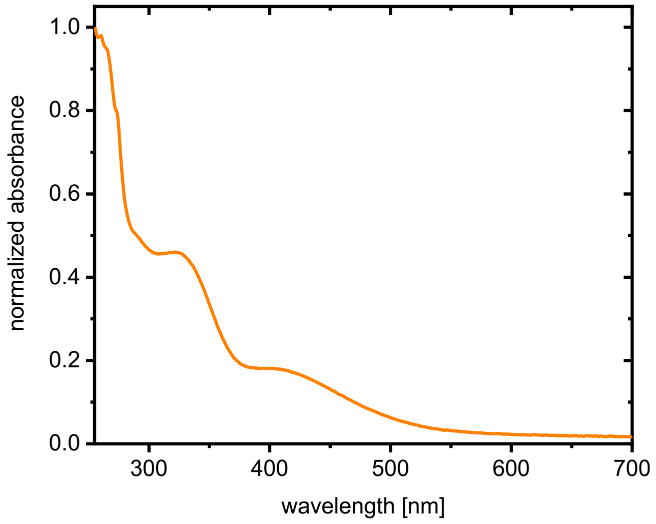

Figure S15. UV-vis absorption spectrum of (CAAC)(CAACH)BeCl in Et_2O at 25 °C. λ_{max} = 325 nm, λ_2 = 404 nm.

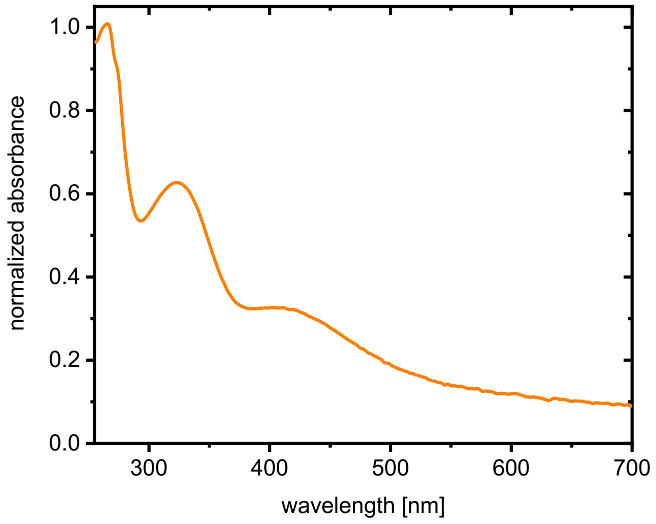

Figure S16. UV-vis absorption spectrum of (CAAC)(CAACH)BeBr in Et_2O at 25 °C. λ_{max} = 266 nm, λ_2 = 325 nm, λ_3 = 425 nm.

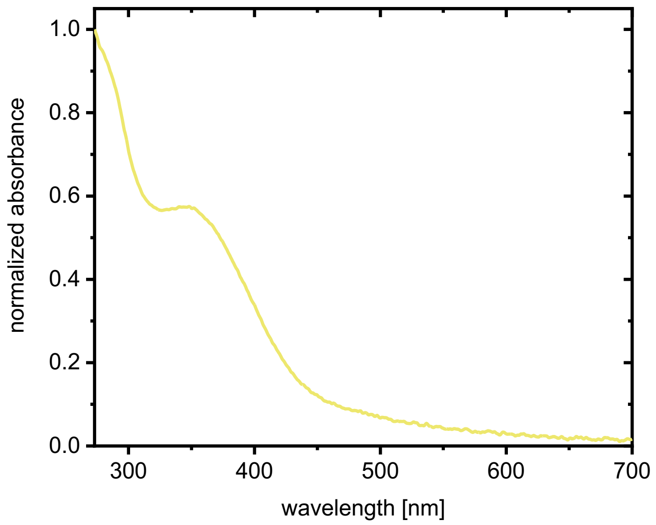

Figure S17. UV-vis absorption spectrum of [(CAAC)(CAACH)Be]$^{\bullet}$ in Et_2O at 25 °C recorded in a normal cuvette.

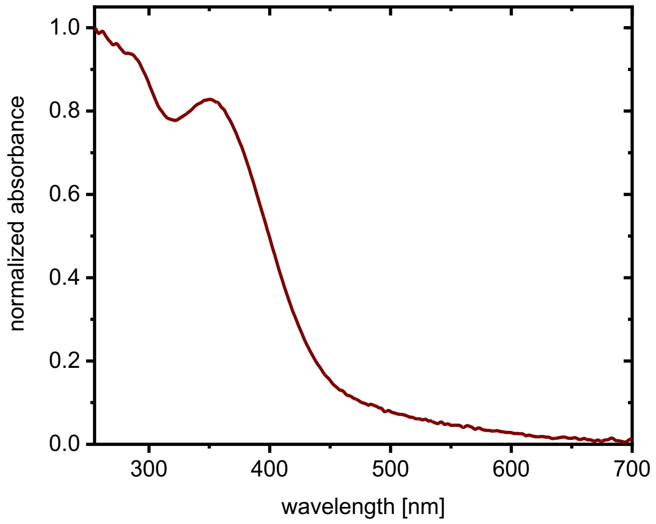

Figure S18. UV-vis absorption spectrum of [(CAAC)(CAACH)Be]$^{\bullet}$ in Et_2O at 25 °C recorded in a silanized cuvette. λ_{max} = 350 nm.

X-ray crystallographic details

The crystal data of (CAAC)(CAACH)BeBr were collected on a Bruker D8 Quest diffractometer with a CMOS area detector and multi-layer mirror monochromated $Mo_{K\alpha}$ radiation, those of $(CAAC)BeBr_2$ were collected on a Bruker X8-APEX II diffractometer with a CCD area detector and multi-layer mirror monochromated $Mo_{K\alpha}$ radiation. The crystal data of $[(CAAC)(CAACH)Be]^{\bullet}$ and (CAAC)(CAACH)BeCl were collected on a XtaLAB Synergy, Dualflex diffractometer with a HyPix area detector and multi-layer mirror monochromated $Cu_{K\alpha}$ radiation. The structures were solved using the intrinsic phasing method,[5] refined with the ShelXL program[6] and expanded using Fourier techniques. All non-hydrogen atoms were refined anisotropically. Hydrogen atoms were included in structure factor calculations. All hydrogen atoms were assigned to idealized geometric positions.

Crystallographic data have been deposited with the Cambridge Crystallographic Data Center as supplementary publication nos. CCDC-2091667 ((CAAC)(CAACH)BeBr), -2091668 ($(CAAC)BeBr_2$), -2091669 ((CAAC)(CAACH)BeCl) and -2091670 ($[(CAAC)(CAACH)Be]^{\bullet}$). These data can be obtained free of charge from The Cambridge Crystallographic Data Centre *via* www.ccdc.cam.ac.uk/data_request/cif.

Refinement details for (CAAC)(CAACH)BeCl: The entire molecule, except for one 2-*i*PrC_6H_3 group (RESI 3 Dip) is disordered by ca. 180° rotation about the Cl-Be axis. The two parts were refined with FVAR to a 58:42 ratio. 1,2- and 1,3-distances in the molecule core (Be, C1 > C8, C21 > C28) were restrained to similarity with SAME. ADPs in the entire molecule were restrained using SIMU 0.008. Due to the extensive disorder and restraints applied the structure may only be used as proof of connectivity.

Crystal data for (CAAC)(CAACH)BeCl: $C_{40}H_{63}BeClN_2$, $M_r = 616.38$, orange block, 0.362×0.191×0.151 mm^3, monoclinic space group $P2_1/c$, $a = 12.46012(17)$ Å, $b = 17.7416(2)$ Å, $c = 17.7209(3)$ Å, $\beta = 107.0328(15)°$, $V = 3745.76(10)$ $Å^3$, $Z = 4$, $\rho_{calcd} = 1.093$ g·cm^{-3}, $\mu = 1.093$ mm^{-1}, $F(000) = 1352$, $T = 103(5)$ K, $R_1 = 0.1127$, $wR_2 = 0.2376$, 7378 independent reflections [$2\theta \leq 144.228°$] and 734 parameters.

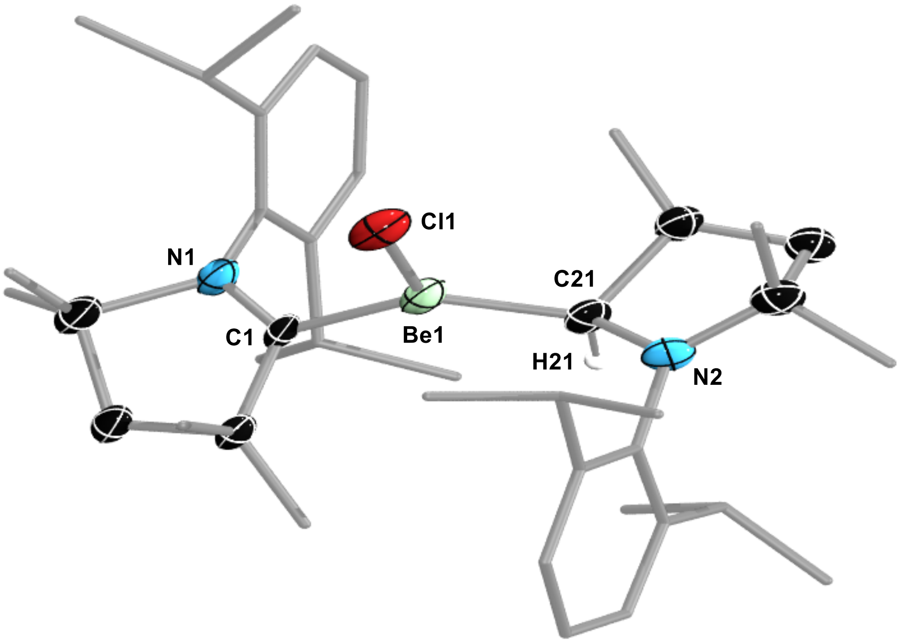

Figure S19. Crystallographically-derived molecular structure of (CAAC)(CAACH)BeCl. Atomic displacement ellipsoids set at 50% probability. Only one of the two rotationally disordered parts of the molecule shown. Ellipsoids of ligand periphery and hydrogen atoms omitted for clarity, except for H21. Due to the extensive disorder and restraints applied no structural parameters may be discussed.

Refinement details for (CAAC)BeBr$_2$: Br1 was modelled as disordered over two positions in a ratio refined to 75:25. The ADPs of both atoms in this disorder were restrained with SIMU 0.005

Crystal data for (CAAC)BeBr$_2$: $C_{20}H_{31}BeBr_2N$, $M_r = 454.29$, colorless block, 0.354×0.238×0.136 mm^3, monoclinic space group $P2_1/c$, $a = 9.385(3)$ Å, $b = 25.134(6)$ Å, $c = 9.901(3)$ Å, $\beta = 114.421(15)°$, $V = 2126.6(10)$ Å^3, $Z = 4$, $\rho_{calcd} = 1.419$ g·cm^{-3}, $\mu = 3.813$ mm^{-1}, $F(000) = 928$, $T = 99(2)$ K, $R_1 = 0.0439$, $wR_2 = 0.0765$, 5105 independent reflections [$2\theta \leq 56.022°$] and 235 parameters.

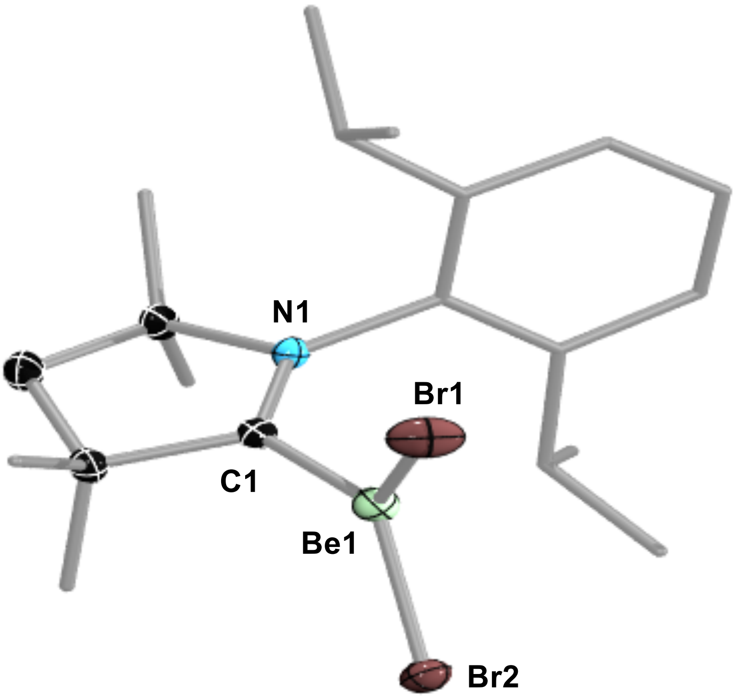

Figure S20. Crystallographically-derived molecular structure of (CAAC)BeBr$_2$. Atomic displacement ellipsoids set at 50% probability. Ellipsoids of ligand periphery and hydrogen atoms omitted for clarity. Selected bond lengths (Å) and angles (°): N1–C1 1.301(3), C1–Be1 1.779(3), Be1–Br1 2.068(3), Be1–Br2 2.076(3), Σ(∠Be1) 358.63(16).

Refinement details for (CAAC)(CAACH)BeBr: The asymmetric unit contains 1.5 benzene molecules. The half molecule is positioned on an inversion center. The other benzene molecule was modelled as twofold disordered in a 73:27 ratio, the rings idealized with AFIX 66 and ADPs restrained with SIMU 0.01 and RIGU 0.01.

Crystal data for (CAAC)(CAACH)BeBr: $C_{49}H_{72}BeBrN_2$, M_r = 778.00, orange block, 0.648×0.584×0.571 mm^3, triclinic space group $P\bar{1}$, a = 10.707(2) Å, b = 13.0332(17) Å, c = 17.635(2) Å, α = 97.38(2)°, β = 102.710(7)°, γ = 106.137(12)°, V = 2258.3(6) Å^3, Z = 2, ρ_{calcd} = 1.144 g·cm^{-3}, μ = 0.943 mm^{-1}, F(000) = 838, T = 100(2) K, R_1 = 0.0283, wR_2 = 0.0627, 8884 independent reflections [$2\theta \leq 52.044°$] and 537 parameters.

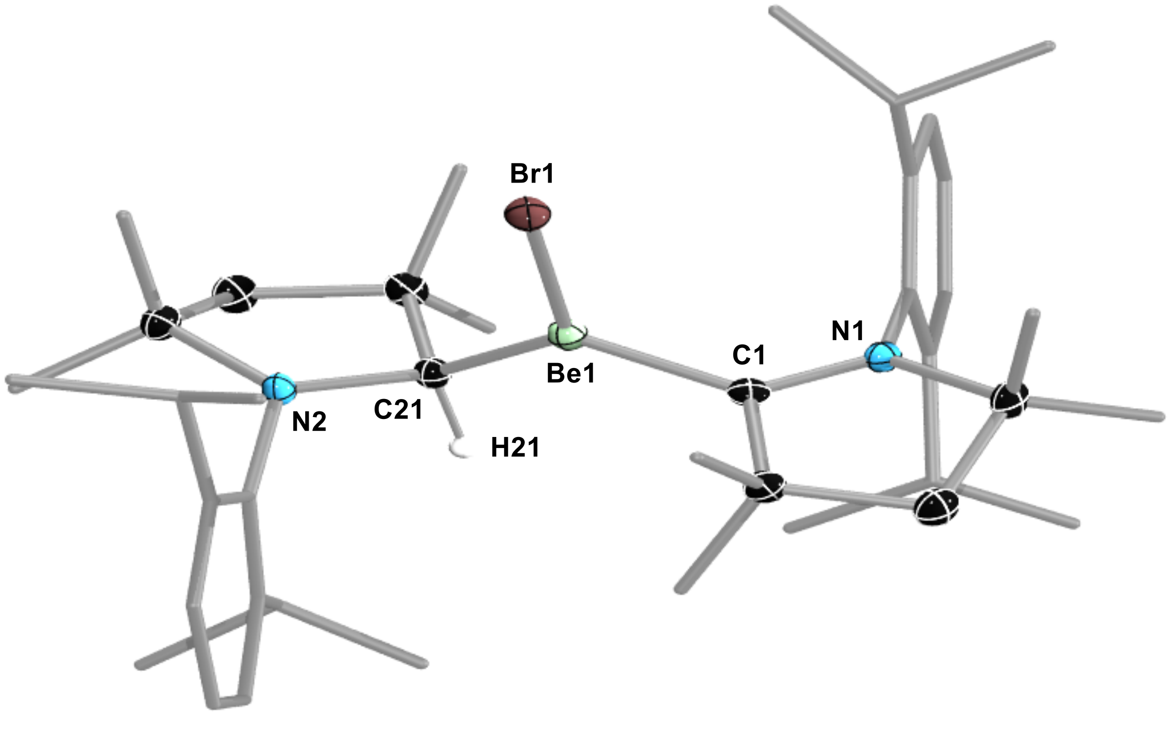

Figure S21. Crystallographically-derived molecular structure of (CAAC)(CAACH)BeBr. Atomic displacement ellipsoids set at 50% probability. Ellipsoids of ligand periphery and hydrogen atoms omitted for clarity, except for H21. Selected bond lengths (Å) and angles (°): N1–C1 1.3060(16), C1–Be1 1.798(2), Be1–Br1 2.1160(17), Be1–C21 1.759(2), C21–N2 1.5148(15), Σ(∠Be1) 359.51(10).

Refinement details for [(CAAC)(CAACH)Be]$^{\bullet}$: The entire molecule is centrosymmetrically disordered. Moreover the CAACH ligand backbone (C21 > C28) is twofold disordered via a mirror plane traversing the (C1,N1,C2) plane. The two parts (PART -1 and -2) of the CAACH ligand were refined with FVAR to a 78:22 ratio. The hydrogen atoms at C21_2 and C21_3 were detected in the difference Fourier map and freely refined. All ADPs within the disordered parts were restrained using SIMU 0.002. No other bond length or angle restraints were applied.

Crystal data for [(CAAC)(CAACH)Be]$^{\bullet}$: $C_{40}H_{63}BeN_2{\cdot}(C_6H_6)_2$, $M_r = 337.19$, orange block, 0.280×0.227×0.182 mm^3, triclinic space group $P\bar{1}$, $a = 10.14520(10)$ Å, $b = 10.66960(10)$ Å, $c = 11.58010(10)$ Å, $\alpha = 73.6480(10)°$, $\beta = 71.0620(10)°$, $\gamma = 78.4260(10)°$, $V = 1129.41(2)$ Å^3, $Z = 1$, $\rho_{calcd} = 1.084$ g·cm^{-3}, $\mu = 0.450$ mm^{-1}, $F(000) = 405$, $T = 99.99(10)$ K, $R_1 = 0.0423$, $wR_2 = 0.1070$, 4457 independent reflections [$2\theta \leq 144.244°$] and 420 parameters.

Computational Details

Geometry optimizations and Hessian calculations were performed for [(CAAC)(CAACH)Be]$^{\bullet}$ and its putative tautomer [(CAAC)$_2$Be]$^{\bullet}$ at the DFT level using the UB3LYP[7] and UBP86[8] functionals with the double-zeta def2-SVP[9] basis set. Dispersion interactions were included using Grimme's D3(BJ) method.[10] Only small structural differences were observed between the optimized geometries at UB3LYP and UBP86. The energy difference between [(CAAC)(CAACH)Be]$^{\bullet}$ and [(CAAC)$_2$Be]$^{\bullet}$ was calculated using the electronic energy in the gas phase corrected by zero-point energy (ZPE). Selected bond orders were estimated using the Mayer bond order (MBO) analysis.[11] A scaling factor of 0.963[12] was used to correct the vibrational frequencies obtained at the UB3LYP-D3(BJ)/def2-SVP level in order to compare the calculated frequencies with the experimental data. Starting from the optimized structure at the UBP86-D3(BJ)/def2-SVP level, a truncated model with Me and *i*Pr groups replaced by hydrogen atoms was constructed. This system, namely [(CAAC')(CAAC'H)Be]$^{\bullet}$, was adopted for further EDA-NOCV[13] calculations, which were conducted at the UBP86-D3(BJ)/TZ2P level of theory. They were based on [(CAAC'H)Be]$^{\bullet}$ and CAAC' as interacting fragments and varied depending on the electronic configuration and multiplicity of the fragments. All DFT calculations were performed using Gaussian 16, revision C.01.[14] The EDA-NOCV calculations were performed using ADF 2019.[15] The MBOs were collected using Multiwfn.[16]

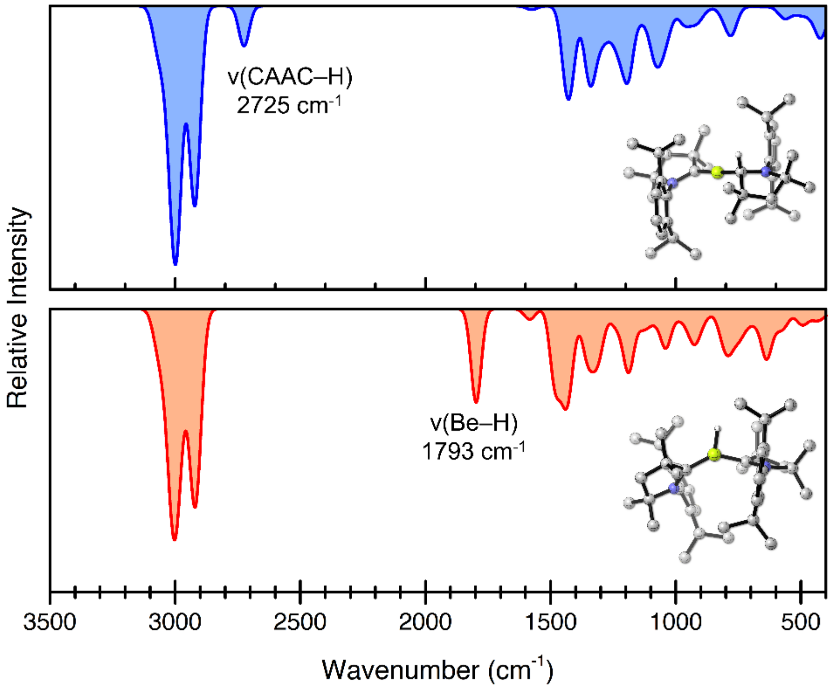

Figure S22. Calculated vibrational frequencies of [(CAAC)(CAACH)Be]$^{\bullet}$ (top) and its putative tautomer [$(CAAC)_2BeH$]$^{\bullet}$ (bottom) at the UB3LYP-D3(BJ)/def2-SVP level. A scaling factor of 0.963 and a Gaussian broadening of 50 cm^{-1} were used.

Table S1. EDA-NOCV results for [(CAAC')(CAAC'H)Be]$^{\bullet}$ considering three distinct fragment interactions at the BP86-D3(BJ)/TZ2P level. D means doublet; S means singlet; and T means triplet. Energy terms are given in kcal/mol.

	Orbital interaction	Interacting Fragments		
		D [(CAAC'H)Be]$^{\bullet}$ ($p_{\perp}$) + S CAAC'	D [(CAAC'H)Be]$^{\bullet}$ (σ) + S CAAC'	D [(CAAC'H)Be]$^{\bullet}$ (σ) + T CAAC'
ΔE_{int}	–	–133.6	–80.27	–129.3
ΔE_{Pauli}	–	102.0	329.4	165.3
ΔE_{elstat}[a]	–	–110.9 (47.1%)	–189.0 (46.1%)	–134.1 (45.5%)
ΔE_{disp}[a]	–	–6.1 (2.6%)	–6.1 (1.5%)	–6.1 (2.1%)
ΔE_{orb}[a]	–	–118.6 (50.3%)	–214.6 (52.4%)	–154.4 (52.4%)
$\Delta E_{orb(1\alpha\beta)}$ [b]	M←CAAC σ donation	–43.4 (36.6%)	–	–
$\Delta E_{orb(2\alpha)}$ [b]	M→CAAC π backdonation	–56.5 (47.7%)	–	–
$\Delta E_{orb(rest)}$ [b]	–	–18.7 (15.7%)	–	–

[a] The values in parentheses give the percentage contribution to the total attractive interactions ΔE_{elstat} + ΔE_{disp} + ΔE_{orb}. [b] The values in parentheses give the percentage contribution to the total orbital interaction ΔE_{orb}.

Cartesian Coordinates

[(CAAC)(CAACH)Be]$^{\bullet}$ – B3LYP-D3(BJ)/def2-SVP

Be	-0.067080000	-0.014779000	-0.303324000
N	-2.518197000	-0.990338000	0.081726000
C	-0.888469000	-2.704246000	-0.084434000
C	-2.256970000	-3.273616000	0.407005000
H	-2.217801000	-3.446761000	1.493193000
H	-2.492529000	-4.241297000	-0.061694000
C	-3.343597000	-2.212855000	0.104315000
C	-2.877203000	0.910512000	1.581830000
C	-3.260996000	2.243485000	1.777155000
C	-4.992375000	0.695976000	-2.737006000
H	-5.090164000	0.179160000	-3.705482000
H	-5.708029000	0.243240000	-2.033547000
H	-5.295674000	1.744481000	-2.888239000
C	-3.552248000	0.593210000	-2.212811000
H	-3.293331000	-0.466264000	-2.165836000
C	-2.563377000	1.242173000	-3.191322000
H	-1.524179000	1.056250000	-2.887108000
H	-2.697763000	0.823855000	-4.202122000
C	-3.325711000	0.084465000	3.938831000
H	-3.448734000	1.082262000	4.390429000
C	-2.340271000	0.113808000	2.762339000
H	-2.202785000	-0.911946000	2.398904000
H	-0.571625000	-0.008273000	4.033609000
C	0.095959000	-2.670111000	1.089065000
H	-0.285002000	-2.018795000	1.889762000
H	0.250984000	-3.673482000	1.520622000
H	1.079757000	-2.294217000	0.775145000
C	-0.289664000	-3.542992000	-1.218372000
H	-0.982414000	-3.617896000	-2.071530000
H	0.649735000	-3.098523000	-1.580963000
C	-4.029326000	-2.550973000	-1.239671000

H	-3.308291000	-2.568933000	-2.070257000
H	-4.489692000	-3.549886000	-1.183027000
C	-4.424542000	-2.149809000	1.184952000
H	-5.022110000	-3.074264000	1.177441000
C	1.059674000	1.186356000	-0.177070000
N	2.426790000	0.974530000	-0.031129000
C	0.833368000	2.699732000	-0.256708000
C	2.242730000	3.230278000	-0.613589000
H	2.344968000	3.255758000	-1.710086000
H	2.418823000	4.251599000	-0.244462000
C	3.257688000	2.221484000	-0.043268000
C	2.948931000	-0.349275000	-0.182798000
C	2.958835000	-0.945645000	-1.473559000
C	3.392092000	-2.271986000	-1.596813000
H	3.398453000	-2.741851000	-2.582705000
C	3.827090000	-2.998912000	-0.490776000
H	4.163555000	-4.031497000	-0.610080000
C	3.828229000	-2.402213000	0.765640000
H	4.162385000	-2.976298000	1.632523000
C	3.380908000	-1.085654000	0.947481000
C	4.807189000	-0.378354000	2.898229000
H	4.815006000	0.170434000	3.853760000
H	5.467518000	0.148957000	2.193331000
H	5.250298000	-1.370850000	3.079909000
C	3.374712000	-0.506129000	2.353996000
H	2.935648000	0.494768000	2.288020000
C	2.503643000	-1.309815000	3.330419000
H	1.456134000	-1.330757000	3.007484000
H	2.539878000	-0.852584000	4.332353000
H	2.849355000	-2.351328000	3.427868000
C	3.720204000	-0.145592000	-3.748637000
H	4.647795000	0.226080000	-3.289640000
H	3.465838000	0.513490000	-4.594319000
H	3.933605000	-1.144000000	-4.162045000

C	2.560289000	-0.193583000	-2.739392000
H	2.315022000	0.835242000	-2.448707000
C	1.308473000	-0.781684000	-3.405561000
H	0.446471000	-0.775914000	-2.725997000
H	1.472602000	-1.822243000	-3.728229000
H	1.037030000	-0.193444000	-4.296805000
C	-0.175044000	3.077305000	-1.348444000
H	0.136124000	2.689556000	-2.330165000
H	-0.273328000	4.173172000	-1.427057000
H	-1.170903000	2.675900000	-1.117338000
C	0.313531000	3.267339000	1.080895000
H	0.939989000	2.970429000	1.932157000
H	-0.703872000	2.902651000	1.275767000
H	0.277142000	4.369568000	1.046249000
C	3.732525000	2.648444000	1.358852000
H	2.907835000	2.681906000	2.081123000
H	4.171662000	3.656298000	1.299990000
H	4.506306000	1.974960000	1.745805000
C	4.493601000	2.072155000	-0.933752000
H	4.222260000	1.883042000	-1.978862000
H	5.136720000	1.248571000	-0.587756000
H	5.085402000	2.999575000	-0.900935000
C	-1.236470000	-1.253465000	-0.573327000
H	-1.388981000	-1.337482000	-1.678536000
C	-2.971503000	0.340729000	0.280295000
H	-3.186428000	2.682188000	2.775075000
C	-3.720149000	3.025152000	0.719085000
H	-4.008174000	4.065651000	0.888424000
C	-3.800025000	2.474347000	-0.557127000
H	-4.150019000	3.092364000	-1.387599000
C	-3.434924000	1.142147000	-0.797810000
H	-2.704776000	2.333024000	-3.257380000
H	-2.962005000	-0.591105000	4.730119000
H	-4.321118000	-0.262107000	3.623228000

C	-0.963563000	0.620623000	3.217871000
H	-0.235459000	0.602239000	2.392428000
H	-1.016907000	1.657207000	3.588477000
H	-0.060721000	-4.565660000	-0.875545000
H	-4.823857000	-1.831858000	-1.481078000
H	-3.990333000	-2.036602000	2.186269000
H	-5.108604000	-1.304878000	1.008722000

[(CAAC)(CAACH)Be]$^{\bullet}$ – BP86-D3(BJ)/def2-SVP

Be	-0.086685000	-0.010061000	-0.338177000
N	-2.508393000	-1.000968000	0.081155000
C	-0.886335000	-2.711012000	-0.179843000
C	-2.248728000	-3.302292000	0.324459000
H	-2.181681000	-3.524462000	1.409813000
H	-2.501268000	-4.255885000	-0.183792000
C	-3.345427000	-2.223723000	0.093421000
C	-2.826635000	0.869901000	1.641745000
C	-3.196928000	2.208425000	1.877455000
C	-5.005326000	0.787604000	-2.666197000
H	-5.128494000	0.293321000	-3.652877000
H	-5.723684000	0.326209000	-1.957572000
H	-5.297339000	1.852265000	-2.787836000
C	-3.555526000	0.649398000	-2.164810000
H	-3.313513000	-0.426153000	-2.131553000
C	-2.562316000	1.295881000	-3.146913000
H	-1.515341000	1.094620000	-2.846259000
H	-2.706022000	0.885021000	-4.168477000
C	-3.213704000	-0.019749000	3.994755000
H	-3.322867000	0.976048000	4.475183000
C	-2.263495000	0.034539000	2.785870000
H	-2.146968000	-0.989043000	2.375911000
H	-0.436186000	-0.144145000	3.986731000
C	0.115177000	-2.674071000	0.984990000
H	-0.269353000	-2.026693000	1.800282000

H	0.294692000	-3.684233000	1.413083000
H	1.097121000	-2.276392000	0.657810000
C	-0.289680000	-3.520963000	-1.340410000
H	-0.994876000	-3.581806000	-2.195837000
H	0.649491000	-3.053467000	-1.702655000
C	-4.075444000	-2.510061000	-1.245052000
H	-3.370253000	-2.504435000	-2.101565000
H	-4.547729000	-3.514095000	-1.214133000
C	-4.391793000	-2.181171000	1.214141000
H	-5.011285000	-3.100907000	1.197882000
C	1.036788000	1.200427000	-0.154136000
N	2.400413000	0.969535000	0.016721000
C	0.823625000	2.720032000	-0.208264000
C	2.252514000	3.245149000	-0.519642000
H	2.387808000	3.279742000	-1.621912000
H	2.428523000	4.269380000	-0.132509000
C	3.244794000	2.216016000	0.070400000
C	2.911726000	-0.350055000	-0.214640000
C	2.881758000	-0.881753000	-1.541867000
C	3.305391000	-2.209942000	-1.743455000
H	3.285868000	-2.630376000	-2.761456000
C	3.770463000	-2.998061000	-0.681467000
H	4.100600000	-4.032676000	-0.863613000
C	3.811839000	-2.461911000	0.609855000
H	4.169212000	-3.084454000	1.445258000
C	3.372524000	-1.147549000	0.871606000
C	4.850759000	-0.485043000	2.803522000
H	4.882228000	0.007983000	3.797942000
H	5.470646000	0.110182000	2.102024000
H	5.336915000	-1.478512000	2.905701000
C	3.398789000	-0.631176000	2.304742000
H	2.925225000	0.368675000	2.291288000
C	2.580824000	-1.505380000	3.273227000
H	1.519578000	-1.559467000	2.967864000

H	2.620875000	-1.081075000	4.298286000
H	2.972945000	-2.542884000	3.327074000
C	3.622290000	0.051728000	-3.778057000
H	4.558934000	0.411158000	-3.306351000
H	3.354262000	0.753632000	-4.595425000
H	3.843589000	-0.931140000	-4.244411000
C	2.468675000	-0.055354000	-2.759276000
H	2.224957000	0.963303000	-2.398124000
C	1.204630000	-0.607470000	-3.442300000
H	0.350076000	-0.653432000	-2.738616000
H	1.368244000	-1.631981000	-3.838317000
H	0.907576000	0.039804000	-4.293677000
C	-0.159067000	3.118433000	-1.322766000
H	0.175132000	2.736863000	-2.309235000
H	-0.254538000	4.223676000	-1.391010000
H	-1.168926000	2.712888000	-1.114932000
C	0.267708000	3.264960000	1.129144000
H	0.885350000	2.963849000	1.996555000
H	-0.755894000	2.878450000	1.299438000
H	0.214809000	4.375327000	1.108365000
C	3.658708000	2.604222000	1.508128000
H	2.796207000	2.603049000	2.200478000
H	4.088896000	3.626913000	1.502038000
H	4.430750000	1.922718000	1.909757000
C	4.514910000	2.064183000	-0.776244000
H	4.276751000	1.884134000	-1.841021000
H	5.139354000	1.222076000	-0.413213000
H	5.121431000	2.990387000	-0.712650000
C	-1.256231000	-1.251366000	-0.637278000
H	-1.451054000	-1.321337000	-1.749126000
C	-2.945200000	0.332483000	0.319657000
H	-3.105970000	2.622501000	2.894718000
C	-3.656034000	3.028834000	0.837813000
H	-3.931010000	4.076351000	1.038879000

C	-3.750371000	2.511914000	-0.460434000
H	-4.100513000	3.161660000	-1.279018000
C	-3.408887000	1.172533000	-0.739623000
H	-2.692082000	2.397533000	-3.204311000
H	-2.824722000	-0.716482000	4.767010000
H	-4.227350000	-0.361002000	3.701733000
C	-0.861994000	0.518795000	3.204205000
H	-0.164515000	0.527263000	2.338984000
H	-0.893483000	1.550547000	3.614945000
H	-0.043703000	-4.556899000	-1.023234000
H	-4.874930000	-1.768682000	-1.440732000
H	-3.917511000	-2.107044000	2.211184000
H	-5.071001000	-1.312210000	1.088041000

$[(CAAC)_2Be]^{\bullet}$ – B3LYP-D3(BJ)/def2-SVP

Be	0.170674000	0.894932000	-0.705000000
N	-2.248928000	0.611602000	0.710825000
C	-1.126159000	2.684750000	1.029792000
C	-2.542794000	2.750484000	1.652501000
H	-3.214407000	3.297722000	0.972359000
H	-2.561536000	3.280761000	2.616944000
C	-3.042714000	1.295845000	1.783938000
C	-3.378679000	0.117267000	-1.430270000
C	-3.979213000	-0.814459000	-2.284800000
C	-3.475795000	-2.810644000	2.380731000
H	-3.054338000	-3.101485000	3.357129000
H	-4.274974000	-2.075740000	2.560156000
H	-3.944751000	-3.705507000	1.940152000
C	-2.384622000	-2.239813000	1.463644000
H	-1.913969000	-1.395886000	1.975359000
C	-1.288633000	-3.284343000	1.205834000
H	-0.464806000	-2.849749000	0.622047000
H	-0.870243000	-3.655528000	2.155267000
C	-4.728824000	2.102997000	-2.259556000

H	-5.111454000	1.627211000	-3.176493000
C	-3.338916000	1.573083000	-1.873576000
H	-2.982863000	2.164360000	-1.024648000
H	-2.325773000	2.846046000	-3.327583000
C	-0.923196000	3.847836000	0.040139000
H	-1.672937000	3.825783000	-0.764327000
H	-1.005060000	4.818059000	0.559809000
H	0.066298000	3.788084000	-0.433053000
C	-0.013992000	2.788686000	2.096869000
H	-0.116344000	2.023827000	2.878091000
H	0.974609000	2.650368000	1.630819000
C	-2.720879000	0.737590000	3.183135000
H	-1.640056000	0.710851000	3.363648000
H	-3.179592000	1.376583000	3.954271000
C	-4.550867000	1.187068000	1.550374000
H	-5.084975000	1.744936000	2.334983000
C	1.123135000	-0.514188000	-1.119539000
N	2.321115000	-0.769297000	-0.631393000
C	0.894607000	-1.413799000	-2.327642000
C	1.971367000	-2.511815000	-2.176975000
H	1.526704000	-3.387521000	-1.680327000
H	2.366494000	-2.849929000	-3.145525000
C	3.075843000	-1.922191000	-1.283742000
C	2.939011000	0.008922000	0.428879000
C	2.772286000	-0.410097000	1.769673000
C	3.446916000	0.289470000	2.778208000
H	3.323528000	-0.023927000	3.816238000
C	4.252245000	1.383904000	2.484003000
H	4.774424000	1.914879000	3.283352000
C	4.364133000	1.815145000	1.167098000
H	4.964872000	2.698292000	0.943487000
C	3.711658000	1.156751000	0.114529000
C	5.299636000	1.825595000	-1.765366000
H	5.335723000	2.185508000	-2.805948000

H	5.801848000	0.850140000	-1.726240000
H	5.894274000	2.526014000	-1.158005000
C	3.842073000	1.773809000	-1.274729000
H	3.247614000	1.172674000	-1.973352000
C	3.255901000	3.196632000	-1.294736000
H	2.212782000	3.190228000	-0.956968000
H	3.274327000	3.595924000	-2.321496000
H	3.842795000	3.878972000	-0.659432000
C	2.473133000	-2.569397000	3.095770000
H	3.390903000	-2.983777000	2.653787000
H	1.773375000	-3.401115000	3.275061000
H	2.736642000	-2.149183000	4.078983000
C	1.817731000	-1.516905000	2.190844000
H	1.457073000	-2.023906000	1.288436000
C	0.593058000	-0.893024000	2.874117000
H	0.054127000	-0.242981000	2.171642000
H	0.893474000	-0.287041000	3.743116000
H	-0.093270000	-1.673261000	3.232174000
C	-0.517261000	-2.001523000	-2.375113000
H	-0.738743000	-2.598706000	-1.482624000
H	-0.621322000	-2.654956000	-3.255986000
H	-1.278452000	-1.214602000	-2.443484000
C	1.115545000	-0.555276000	-3.595495000
H	2.099970000	-0.067812000	-3.603736000
H	0.357326000	0.235308000	-3.664195000
H	1.037185000	-1.199259000	-4.485922000
C	4.282986000	-1.422338000	-2.078354000
H	4.005775000	-0.695664000	-2.851032000
H	4.764617000	-2.279123000	-2.572430000
H	5.022474000	-0.961812000	-1.411439000
C	3.566415000	-2.925501000	-0.239608000
H	2.735694000	-3.366985000	0.324358000
H	4.268053000	-2.461488000	0.468168000
H	4.094754000	-3.741794000	-0.754817000

C	-1.097955000	1.315726000	0.329505000
C	-2.854124000	-0.325567000	-0.182506000
H	-4.381707000	-0.475647000	-3.242027000
C	-4.093086000	-2.159516000	-1.934940000
H	-4.575519000	-2.867668000	-2.613086000
C	-3.587376000	-2.586530000	-0.712759000
H	-3.668580000	-3.640679000	-0.436361000
C	-2.959108000	-1.694092000	0.168267000
H	-1.674668000	-4.154067000	0.650469000
H	-4.682437000	3.187653000	-2.448775000
H	-5.467805000	1.927240000	-1.462764000
C	-2.339590000	1.787983000	-3.019289000
H	-1.319526000	1.528108000	-2.702057000
H	-2.605296000	1.182245000	-3.901349000
H	-0.026668000	3.778169000	2.586698000
H	-3.116622000	-0.277293000	3.316078000
H	-4.841650000	1.604064000	0.577917000
H	-4.890930000	0.141557000	1.585168000
H	0.792151000	1.825523000	-1.519836000

$[(CAAC)_2Be]^{\bullet}$ – BP86-D3(BJ)/def2-SVP

Be	0.263121745	0.218132683	-0.943776511
N	-2.449315418	0.886175038	-0.259611712
C	-1.411574491	2.292974233	-1.826627258
C	-2.894268287	2.726211118	-1.644651668
H	-3.507149206	2.326090146	-2.480570743
H	-3.022893284	3.827435852	-1.644269073
C	-3.371280830	2.088062515	-0.324849742
C	-3.514366654	-1.284977301	-0.651558484
C	-4.077732987	-2.468396491	-0.138513203
C	-3.098308451	0.765041603	3.756804448
H	-2.584827128	1.513635319	4.396063795
H	-4.014790528	1.235202889	3.344888115
H	-3.424028160	-0.067534659	4.415645469

C	-2.161807069	0.266256591	2.640824135
H	-1.794929011	1.139959451	2.070419216
C	-0.926715836	-0.435987548	3.237064017
H	-0.216019149	-0.716968496	2.435101717
H	-0.398030193	0.233440722	3.947168555
C	-4.857740781	-1.398599594	-2.824009424
H	-5.046832217	-2.492566133	-2.817981566
C	-3.517715288	-1.039772968	-2.158477485
H	-3.346014733	0.042815202	-2.316008790
H	-2.345878200	-1.586189753	-3.932764880
C	-1.127288831	1.936220395	-3.302948172
H	-1.807555709	1.135966279	-3.660870434
H	-1.270114829	2.828032758	-3.951615761
H	-0.084024351	1.577042925	-3.406700780
C	-0.424461538	3.405515423	-1.404874578
H	-0.618711923	3.770818649	-0.377352592
H	0.610710234	3.011077285	-1.442099551
C	-3.123742826	3.024082346	0.876531841
H	-2.046815794	3.248033862	0.997234526
H	-3.660384120	3.982683228	0.721053215
C	-4.850679969	1.687123964	-0.353205176
H	-5.474013197	2.600361655	-0.439863242
C	1.108493513	-1.061995767	-0.106991173
N	2.372455069	-0.857157075	0.337905603
C	0.836492703	-2.563265272	0.043266061
C	1.844246743	-3.018355589	1.128943568
H	1.345523116	-2.976341033	2.119672918
H	2.192402510	-4.060687659	0.980343717
C	3.013163302	-2.009683427	1.096981262
C	3.105554475	0.375931222	0.139766295
C	2.964982772	1.436976586	1.074086408
C	3.739723665	2.600397110	0.893161765
H	3.637564410	3.428700266	1.611655879
C	4.623319493	2.725096671	-0.183782729

H	5.225520028	3.639285823	-0.305110902
C	4.717481822	1.688966030	-1.120444452
H	5.381681292	1.804677514	-1.991066971
C	3.960139748	0.508600721	-0.993616012
C	5.460185266	-0.982957853	-2.430776222
H	5.449005868	-1.791261284	-3.191781262
H	5.981402631	-1.366527748	-1.531165374
H	6.075691433	-0.156367114	-2.843911251
C	4.024933798	-0.511033349	-2.129479631
H	3.422224195	-1.386761370	-1.824036935
C	3.369900736	0.057091875	-3.405962578
H	2.343026993	0.405463017	-3.173990852
H	3.324718524	-0.721957579	-4.196396222
H	3.953892281	0.913368928	-3.805915801
C	2.586305120	1.728407198	3.582573368
H	3.454824689	1.075654890	3.802164993
H	1.840427671	1.592956343	4.393991007
H	2.936661206	2.780599426	3.634305736
C	1.955377670	1.407486597	2.215740814
H	1.534041452	0.386766655	2.262435414
C	0.783687693	2.360600440	1.913432964
H	0.256839353	2.044266747	0.989822869
H	1.139264112	3.401725456	1.765494585
H	0.054492027	2.365902267	2.750012981
C	-0.599218621	-2.930018973	0.423624380
H	-0.883050776	-2.509735289	1.404685132
H	-0.711843944	-4.033179967	0.478137574
H	-1.320107791	-2.550627461	-0.321663566
C	1.151884989	-3.205335079	-1.335823826
H	2.181000328	-2.979675378	-1.677758585
H	0.455309926	-2.825480007	-2.109100486
H	1.038507705	-4.309115739	-1.275220331
C	4.261918420	-2.560064808	0.390832853
H	4.037295378	-2.969520996	-0.611114525

H	4.691876523	-3.378025194	1.003684138
H	5.034797139	-1.774098478	0.286865928
C	3.420329211	-1.561822200	2.509503509
H	2.549328331	-1.200215277	3.088512322
H	4.181776113	-0.756799138	2.473431084
H	3.857718550	-2.423340753	3.054069762
C	-1.280063890	1.014963551	-0.961089074
C	-2.927189462	-0.360566031	0.262734917
H	-4.537912931	-3.190506474	-0.830193377
C	-4.068930653	-2.741196359	1.237191537
H	-4.524847545	-3.667942359	1.619633226
C	-3.467727088	-1.836752535	2.118456868
H	-3.439445210	-2.064968161	3.195887148
C	-2.878727939	-0.642547124	1.651936322
H	-1.207499401	-1.357546552	3.789671647
H	-4.857279339	-1.075320979	-3.885613070
H	-5.713346837	-0.908204687	-2.314925564
C	-2.344893930	-1.773823835	-2.838496180
H	-1.377997136	-1.412533360	-2.432352641
H	-2.409631358	-2.870778638	-2.677056902
H	-0.507761446	4.273223763	-2.094547053
H	-3.495563928	2.580972072	1.819353187
H	-5.085060510	1.031969191	-1.213776502
H	-5.146855713	1.156987845	0.574366869
H	1.157110085	0.896891407	-1.765610475

[(CAAC')(CAAC'H)Be]$^{\bullet}$

Be	0.000000000	0.000000000	0.000000000
N	-1.047600000	2.312700000	-0.759200000
C	1.280200000	2.490600000	-0.343100000
C	0.733700000	3.786600000	-1.037300000
H	1.014200000	3.782900000	-2.111100000
H	1.165800000	4.708200000	-0.595400000
C	-0.817000000	3.767900000	-0.917100000

C	-2.444200000	0.992200000	-2.289900000
C	-3.611500000	0.241300000	-2.530400000
C	-0.020800000	1.728100000	0.107100000
H	-0.196000000	2.033900000	1.181800000
C	-2.259600000	1.610200000	-1.011300000
H	-3.754100000	-0.234400000	-3.514200000
C	-4.582500000	0.069300000	-1.534300000
H	-5.484100000	-0.530200000	-1.737000000
C	-4.392600000	0.653400000	-0.275600000
H	-5.151500000	0.507500000	0.510300000
C	-3.248800000	1.429100000	0.004300000
H	1.908400000	2.710200000	0.494800000
H	-1.309300000	4.153200000	-1.785500000
H	-1.115000000	4.354500000	-0.073300000
H	-1.697100000	1.063100000	-3.052600000
H	-3.145700000	1.876700000	0.970700000
H	1.828100000	1.910500000	-1.056000000
C	0.000000000	-1.661700000	0.000000000
N	1.159700000	-2.434400000	0.000000000
C	-1.198600000	-2.618600000	0.072600000
C	-0.544500000	-3.932700000	0.582100000
H	-0.551000000	-3.927300000	1.693200000
H	-1.089300000	-4.840600000	0.252600000
C	0.923100000	-3.919200000	0.094200000
C	2.416800000	-1.793200000	0.255000000
C	2.661300000	-1.238600000	1.550300000
C	3.862800000	-0.535200000	1.764300000
H	4.061500000	-0.103000000	2.757800000
C	4.817700000	-0.393100000	0.747700000
H	5.751800000	0.157500000	0.939300000
C	4.575700000	-0.954700000	-0.510300000
H	5.322400000	-0.835300000	-1.311200000
C	3.376700000	-1.644100000	-0.786400000
H	-1.941100000	-2.272500000	0.761000000

H	-1.667300000	-2.735600000	-0.882100000
H	1.594800000	-4.375600000	0.790900000
H	1.020200000	-4.418900000	-0.846900000
H	3.215900000	-2.032200000	-1.770400000
H	1.992900000	-1.370200000	2.375400000

References

[1] M. Müller, F. Pielnhofer, M. R. Buchner, *Dalton Trans*. **2018**, *47*, 12506-12510.

[2] A. Paparo, C. Jones, *Chem. Asian J.* **2019**, *14*, 486-490.

[3] V. Lavallo, Y. Canac, C. Präsang, B. Donnadieu, G. Bertrand, *Angew. Chem. Int. Ed. Engl*. **2005**, 44, 5705-5709.

[4] M. Arrowsmith, H. Braunschweig, M. A. Celik, T. Dellermann, R. D. Dewhurst, W. C. Ewing, K. Hammond, T. Kramer, I. Krummenacher, J. Mies, K. Radacki, J. K. Schuster, *Nature Chem*. **2016**, *8*, 890-894.

[5] G. Sheldrick, *Acta Cryst.* **2015**, *A71*, 3–8.

[6] G. Sheldrick, *Acta Cryst.* **2008**, *A64*, 112–122.

[7] a) S. H. Vosko, L. Wilk, M. Nusair, *Can. J. Phys.* **1980**, *58*, 1200–1211; b) C. Lee, W. Yang, R. G. Parr, *Phys. Rev. B* **1988**, *37*, 785–789; c) A. D. Becke, *J. Chem. Phys.* **1993**, *98*, 5648–5652; d) P. J. Stephens, F. J. Devlin, C. F. Chabalowski, M. J. Frisch, *J. Phys. Chem.* **1994**, *98*, 11623–11627.

[8] a) J. P. Perdew, *Phys. Rev. B* **1986**, *33*, 8822–8824; b) A. D. Becke, *Phys. Rev. A* **1988**, *38*, 3098–3100.

[9] F. Weigend, R. Ahlrichs, *Phys. Chem. Chem. Phys.* **2005**, *7*, 3297–3305.

[10] a) S. Grimme, J. Antony, S. Ehrlich, H. Krieg, *J. Chem. Phys.* **2010**, *132*, 154104; b) S. Grimme, S. Ehrlich, L. Goerigk, *J. Comput. Chem.* **2011**, *32*, 1456–1465.

[11] a) I. Mayer, *Chem. Phys. Lett.* **1983**, *97*, 270–274; b) I. Mayer, *Int. J. Quantum Chem.* **1984**, *26*, 151–154.

[12] CCCBDB listing of precalculated vibrational scaling factors. NIST webpage: https://cccbdb.nist.gov/vibscalejust.asp

[13] M. P. Mitoraj, A. Michalak, T. Ziegler, *J. Chem. Theory Comput.* **2009**, *5*, 962–975.

[14] M. J. Frisch, G. W. Trucks, H. B. Schlegel, G. E. Scuseria, M. A. Robb, J. R. Cheeseman, G. Scalmani, V. Barone, B. Mennucci, G. A. Petersson, H. Nakatsuji, M. Caricato, X. Li, H. P. Hratchian, A. F. Izmaylov, J. Bloino, G. Zheng, J. L. Sonnenberg, M. Hada, M. Ehara, K. Toyota, R. Fukuda, J. Hasegawa, M. Ishida, T. Nakajima, Y. Honda, O. Kitao, H. Nakai, T. Vreven, J. A. Montgomery Jr., J. E. Peralta, F. Ogliaro, M. Bearpark, J. J. Heyd, E. Brothers, K. N. Kudin, V. N. Staroverov, R. Kobayashi, J. Normand, K. Raghavachari, A. Rendell, J. C. Burant, S. S. Iyengar, J. Tomasi, M. Cossi, N. Rega, J. M. Millam, M. Klene, J. E. Knox, J. B. Cross, V. Bakken, C. Adamo, J. Jaramillo, R. Gomperts, R. E. Stratmann, O. Yazyev, A. J. Austin, R. Cammi, C.

Pomelli, J. W. Ochterski, R. L. Martin, K. Morokuma, V. G. Zakrzewski, G. A. Voth, P. Salvador, J. J. Dannenberg, S. Dapprich, A. D. Daniels, Ö. Farkas, J. B. Foresman, J. V Ortiz, J. Cioslowski, D. J. Fox, *Gaussian 16, Revision C.01*, Gaussian, Inc., Wallingford CT, **2016**.

[15] G. te Velde, F. M. Bickelhaupt, E. J. Baerends, C. Fonseca Guerra, S. J. A. van Gisbergen, J. G. Snijders, T. Ziegler, *J. Comput. Chem.* **2001**, *22*, 931–967.

[16] T. Lu, F. Chen, *J. Comput. Chem.* **2012**, *33*, 580–592.
